# Experimental, spectroscopic, thermodynamic, and DFT study of a novel cyanomethylchrome nopyridinecarbonitrile (CCPC)

**DOI:** 10.1038/s41598-026-41126-w

**Published:** 2026-03-27

**Authors:** Al-Shimaa Badran, Magdy A. Ibrahim, Shimaa Abdel Halim

**Affiliations:** Department of Chemistry, Faculty of Education, Ain Shams University, Roxy, 11711 Cairo Egypt

**Keywords:** Chromones, Chromeno[4,3-*b*]pyridine, DFT calculations, Thermokinetics, UV–Vis, Drug-likeness, Chemical biology, Chemistry

## Abstract

A novel heterocyclic system, 5-cyanomethylchromeno[4,3-*b*]pyridine-3-carbonitrile (**CCPC**, **3**), was synthesized *via* the reaction of 3-(6,8-dimethylchromonyl)acrylonitrile (**1**) with cyanoacetamide (**2**) under basic conditions. The transformation proceeds through a cascade mechanism involving Michael addition, γ-pyrone ring opening, and double recyclization. The structure of **CCPC** was confirmed by analytical and spectral data. Density functional theory (DFT/B3LYP/6-311 + + G(d, p)) was used to optimize its molecular geometry and investigate electronic properties, global reactivity descriptors, molecular electrostatic potential, and intramolecular charge transfer characteristics. Thermokinetic behavior was evaluated using KiSThelP, while UV–Vis spectra were simulated *via* TDDFT, showing solvent-dependent absorption shifts. SwissADME analysis indicated favorable drug-likeness according to Lipinski’s and Veber’s rules. Overall, the combined experimental and computational study provides insight into the structure, reactivity, stability, and potential applications of **CCPC**.

**Supplementary Information:**

The online version contains supplementary material available at 10.1038/s41598-026-41126-w.

## Introduction

Chromone is a versatile heterocyclic scaffold characterized by a benzopyran-4-one core structure and serves as a key building block in a wide range of natural products and synthetic compounds, particularly within the fields of medicinal chemistry and material science^1–3^. Naturally derived chromone analogs are abundantly found in plants and are associated with a wide spectrum of pharmacological activities^4,5^. These biological properties include anti-inflammatory^6^, antimicrobial^7^, antiangiogenic^8^, antioxidant, anticancer^9^, and antidiabetic effects^10^.

Due to their structural adaptability, chromones have emerged as promising candidates in drug development. Moreover, their distinctive photophysical behavior has made them valuable in material science applications such as fluorescence-based sensing and organic electronic devices^11–13^. The structural simplicity of chromone, combined with its chemical reactivity, allows for extensive modification, enabling the generation of a broad library of derivatives with tailored properties^14–17^. Density Functional Theory (DFT) is a modelling technique recognized for its ability to accurately calculate the physicochemical properties of molecules while maintaining low computational costs^18,19^.

Theoretical research using the DFT approach, computational studies, and electrical, optical, and photoelectrical characteristics is very important to find new drug candidates and understanding the electrical properties of different molecular structures^20–23^. The DFT/B3LYP/6-311 + + G(d, p) level of theory was used for the investigation due to its well-established balance between accuracy and computational efficiency. The B3LYP functional is a hybrid functional that combines Hartree-Fock exchange with DFT exchange-correlation, which provides a good approximation for a wide range of molecular systems. The 6-311 + + G(d, p) basis set includes polarization and diffuse functions, which are crucial for accurately describing electron density in regions far from the nuclei and for modeling non-covalent interactions and anionic species. Moreover, this level of theory performs the better correlations when the predicted spectra are compared with the corresponding experimental ones. The nonlinear optical (NLO) effect is at the forefront of current research because chromeno[4,3-*b*]pyridines are crucial for providing the essential functions of frequency shifting, optical modulation, optical switching, optical logic, and optical memory for emerging technologies in fields like telecommunications, signal processing, and optical interconnections^24^. Molecular electrostatic potential (MEP), represented as a color-coded surface that reflects molecular size, shape, and charge distribution, is a powerful tool for analyzing the structural and physicochemical properties of molecules, including biomolecules and pharmaceutical compounds^25^.

In this study, we aimed to explore the chemical reactivity of an electron-deficient chromone-linked acrylonitrile toward cyanoacetamide under mild basic conditions. The primary goal was to synthesize and characterize a novel compound, **CCPC**, and to investigate its physicochemical and electronic properties. Density Functional Theory (DFT) calculations at the B3LYP/6-311 + + G(d, p) level were utilized to optimize the molecular geometries of the target compound and to compute key global reactivity descriptors, as well as their thermodynamic and kinetic stability parameters. Electronic absorption properties were examined via UV–Vis spectral simulations using time-dependent DFT (TDDFT-CAM-B3LYP/6-311 + + G(d, p)), which revealed solvent-induced red and blue shifts in the absorption maxima (λ_max_) and spectral intensities. The electronic nature of the excited states and the corresponding transitions were thoroughly analyzed. Natural Bond Orbital (NBO) analysis was carried out to investigate intramolecular charge transfer processes in **CCPC**. Furthermore, vibrational (IR) and nuclear magnetic resonance (NMR) spectra were theoretically predicted and compared with experimental results to validate the structural identity of the synthesized compound. Bioavailability parameters were evaluated through ADME analysis and molecular electrostatic potential (MEP) maps were constructed to identify electrophilic and nucleophilic sites, thereby supporting the proposed reaction mechanism and spectroscopic interpretations.

This work reports the first synthesis of the heterocyclic system 5-cyanomethylchromeno [4,3-*b*]pyridine-3-carbonitrile (**CCPC**, **3**), generated through an unprecedented cascade sequence including *Michael* addition, γ-pyrone ring opening, and double recyclization. The study is rationalized by integrating experimental chemistry with a comprehensive computational approach including DFT optimization, global reactivity descriptors, MEP analysis, NBO charge-transfer evaluation, NLO properties, thermo kinetic profiling via Kist help, and TDDFT-simulated UV–Vis spectra. This combined approach not only verifies the structure of the newly synthesized compound but also provides deep insights into its electronic characteristics, stability, and potential functional applications. Furthermore, SwissADME analysis confirms that **CCPC** satisfies Lipinski’s and Veber’s criteria, supporting its promise as a drug-like lead candidate and underscoring the novelty and scientific relevance of the study.

## Experimental

### General

A Perkin-Elmer CHN-2400 analyzer was used to conduct elemental microanalyses. A digital Stuart SMP3 device was used to measure melting points. Using KBr discs, infrared spectra were recorded on an FTIR Nicolet IS10 spectrophotometer (cm-1). Mercury-300BB was used to measure the ^1^H NMR (300 MHz) and ^13^C NMR (75 MHz) spectra. The solvent used was DMSO-*d*_*6*_, and the internal standard was TMS (δ). The GC-2010 Shimadzu gas chromatography mass spectrometry equipment (70 eV) was used to obtain mass spectra. 3-(6,8-Dimethylchromonyl)acrylonitrile (**1**) was prepared according to the published method^26^.

### Synthesis and characterization of compounds

#### 4.2. 5-(Cyanomethyl)-7,9-dimethyl-2-oxo-1,5-dihydro-2 H-chromeno[4,3-b]pyridine-3-carbonitrile (CCPC, 3)

A mixture of 3-(6,8-dimethylchromonyl)acrylonitrile (**1**) (0.68 g, 3 mmol) and cyanoacetamide (**2**) (0.25 g, 3 mmol) in absolute ethanol (25 mL) containing TEA (0.1 mL) was heated under reflux for 2 h. The yellow crystals deposited during heating were filtered off, air dried and crystallized from MeOH to give compound **3** as pale-yellow crystals, yield 0.56 g (65%), mp > 300 °C. IR (KBr, cm^− 1^): 3242 (NH), 3027 (CH_arom_.), 2971, 2934 (CH_aliph_.), 2246, 2213 (2 C ≡ N), 1652 (C = O), 1577 (C = C). ^1^H NMR (DMSO-*d*_6_, δ): 2.30 (s, 3 H, CH_3_), 2.36 (s, 3 H, CH_3_), 3.17 (d, 2 H, *J* = 6.3 Hz, CH_2_CN), 5.47 (t, 1H, *J* = 6.3 Hz, H-5), 7.29 (s, 1H, H-8), 7.53 (s, 1H, H-10), 8.34 (s, 1H, H-4), 12.62 (bs, 1H, NH exchangeable with D_2_O). ^13^C NMR (DMSO-*d*_6_, δ): 17.5 (CH_3_), 20.4 (CH_3_), 22.3 (CH_2_), 70.4 (C-5), 108.2 (C-3), 112.8 (C-4a), 116.3 (C ≡ N), 117.2 (C ≡ N), 119.8 (C-10a), 125.4 (C-10b), 127.7 (C-9), 128.5 (C-7), 130.9 (C-8), 133.7 (C-10), 145.4 (C-4), 148.6 (C-6a), 162.4 (C-2). Mass spectrum, *m/z* (*I*%): 291 (M^+^, 57), 251 (M^+^ - CH_2_CN; 100), 223 (43), 172 (14), 122 (50), 108 (12), 94 (35), 77 (23), 65 (19). Analysis Calcd for C_17_H_13_N_3_O_2_ (291.31); C, 70.09; H, 4.50; N, 14.42%. Found: C, 69.87; H, 4.41; N, 14.36%.

### Computational methods

Computational chemistry calculations for the synthesized compounds were carried out using the GAUSSIAN 09 W software, applying DFT at the B3LYP level with the 6-311 + + G(d, p) basis set. This method is well-suited for evaluating the stability and reactivity of molecules similar to those investigated in this study^27–29^. Visual representations were generated using Gauss-View 5.0^30,31^. Geometry optimizations were performed without imposing any symmetry constraints. Quantum chemical calculations provided results such as molecular electrostatic potential (MEP) maps and optimized geometries along with molecular orbital (MO) energies.

Additionally, the chemical shifts of the ^1^H and ^13^C NMR spectra were calculated using the Gauge-Including Atomic Orbital (GIAO) method at the B3LYP/6-311 + + G(d, p) level, and the results were compared with experimental values to validate the molecular structure^32^. Vibrational frequencies were also computed based on the optimized geometry to further support structural characterization.

The current work concentrated on the total dipole moment *µ*_*tot*_, the average polarizability *α*_*tot*_ and the first hyperpolarizability *β*_*tot*_, using the x, y and z components as follows^33–36^:

1 $$\mu ={\text{ }}{({\mu ^2}_x+{\mu ^2}_{y}+{\mu ^2}_{z})^{1/2}},$$

2 $$\left\langle \alpha \right\rangle =1/3({\alpha _{xx}}+{\alpha _{yy}}+{\alpha _{zz}}),$$

3 $$\Delta \alpha ={\text{ }}{({({\alpha _{xx}} - {\alpha _{yy}})^2}+{\text{ }}{({\alpha _{yy}} - {\alpha _{zz}})^2}+{\text{ }}{({\alpha _{zz}} - {\alpha _{xx}})^2}/2)^{1/2}}$$

4 $$\left\langle \beta \right\rangle ={\text{ }}{({\beta ^2}_{x}+{\beta ^2}_{y}+{\beta ^2}_{z})^{1/2}},$$

Where

$${\beta _x}={\beta _{xxx}}+{\beta _{xyy}}+{\beta _{xzz}},~~~~~~~~~~{\beta _y}={\beta _{yyy}}+{\beta _{xxy}}+{\beta _{yzz}},~~~~~~~~~~~~~~~~~{\beta _z}={\beta _{zzz}}+{\beta _{xxz}}+{\beta _{yyz}}.~~~~~~~$$

Intrinsic Reaction Coordinate (IRC) calculations were performed using 20 points in both the forward and reverse directions, with a step size of 0.1 amu^1/2^ Bohr. The kinetic and statistical thermodynamics software package KiSThelP^37^ was employed to determine the unimolecular rate coefficients (k_uni_, in s^− 1^) for **CCPC** in the gas phase and various solvents. To account for quantum mechanical tunneling effects, the tunneling correction factor *χ(T)* was estimated using the one-dimensional Eckart tunneling model (Eck)^38^, which has been widely applied in prior studies^39^:

5 $$\:{}_{Eckart}\left(T\right)=\frac{\mathrm{e}\mathrm{x}\mathrm{p}\left(\varDelta\:{H}_{f}^{\ne\:,0K}/{k}_{B}T\right)}{{k}_{B}T}{\int\:}_{o}^{\infty\:}p\left(E\right)\mathrm{e}\mathrm{x}\mathrm{p}\left(-E/{k}_{B}T\right)\:dE$$

Where, Δ*H*_*f*_^≠,0 K^ represents the zero-point energy (ZPE)-corrected activation enthalpy in the forward direction, while *p(E)* denotes the transmission probability associated with the corresponding one-dimensional potential energy barrier at energy *E*. Orbital interactions, atomic charges, and their influence on molecular structure and stability were analyzed using the Natural Bond Orbital (NBO) method^40^. Additionally, the electronic absorption spectra (EAS) of the studied compounds were investigated using time-dependent density functional theory (TD-DFT) combined with the Coulomb-Attenuating Method (CAM-B3LYP), based on geometries optimized at the B3LYP/6-311 + + G(d, p) level in the gas phase^41,42^.

## Results and discussion

### Characterization of the synthesized compounds

**Scheme 1:**
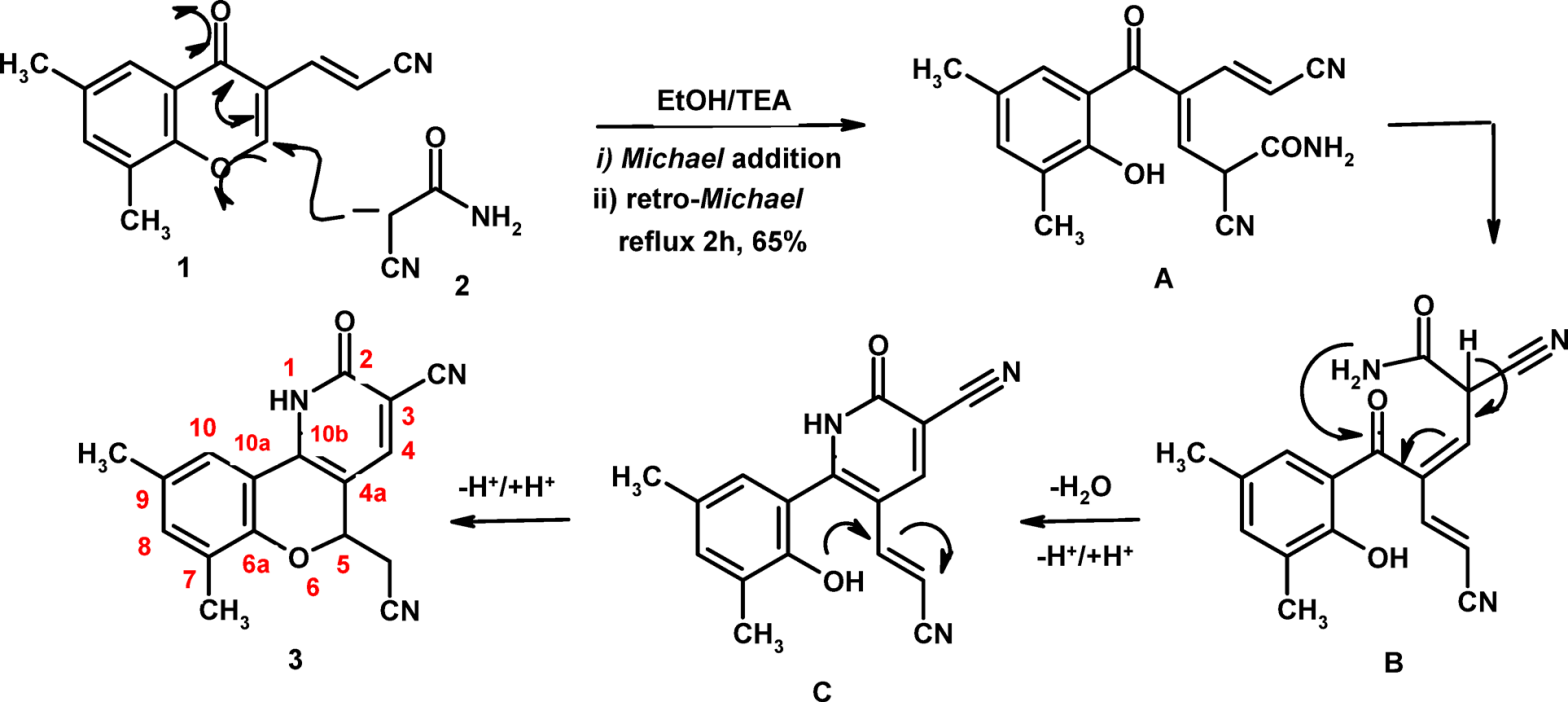
Reaction of acrylonitrile **1** with cyanoacetamide.

The chemical reactivity of 3-(6,8-dimethylchromonyl)acrylonitrile (**1**) toward cyanoacetamide (**2**) was investigated in boiling ethanol using triethylamine (TEA) as a basic catalyst. This reaction yielded 5-(cyanomethyl)-7,9-dimethyl-2-oxo-1,5-dihydro-2*H*-chromeno[4,3-b]pyridine-3-carbonitrile (**3**, **CCPC**), as outlined in Scheme 1. The reaction proceeds *via* a cascade mechanism initiated by a *Michael* addition of the deprotonated cyanoacetamide at the C-2 position of the γ-pyrone ring, followed by a *retro-Michael* step leading to ring opening and formation of intermediate **A**. This intermediate undergoes free rotation around a single bond (intermediate **B**), which is then followed by intramolecular cyclocondensation (intermediate **C**) and subsequent cycloaddition, ultimately furnishing the final product **3**, as depicted in Scheme 1. The electron-deficient sites in substrate **1** were further evaluated through theoretical calculations to support the proposed reaction mechanism. The computational results confirmed that the C-2 position of the γ-pyrone moiety is the most electron-deficient center, rendering it the most favorable site for nucleophilic attack in the initial *Michael* addition step.

**Scheme 2:**
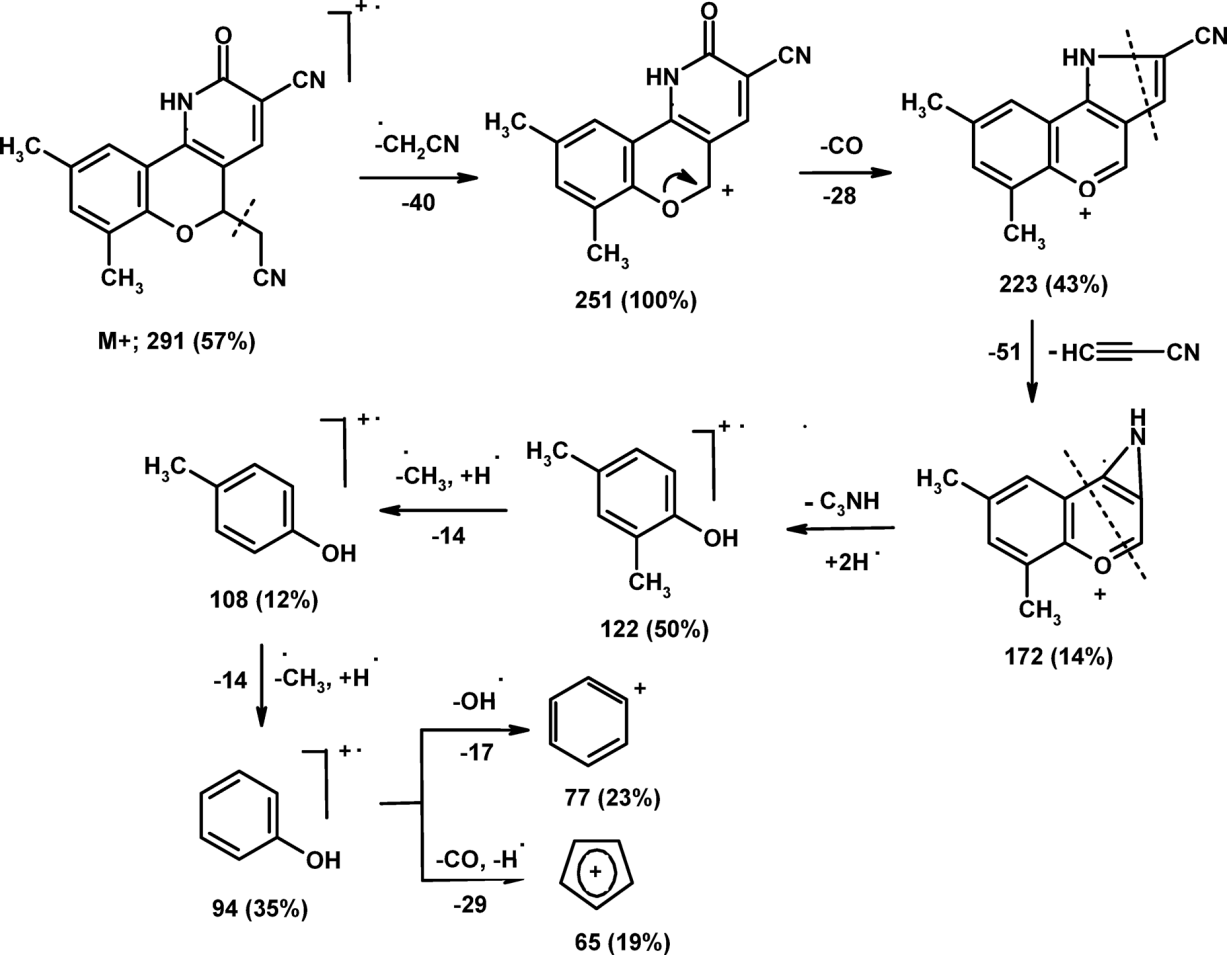
Mass fragmentation patterns of compound **3 (CCPC)**.

Structure of product **3** was elucidated based on spectral results. The mass spectrum displayed the molecular ion peak at *m/z* 291 corresponding to the suggested molecular formula (C_17_H_13_N_3_O_2_) and the base peak at *m/z* 251, which attribute to loss of a CH_2_CN fragment from the molecular ion. The detailed fragmentation pathway of compound **3** is illustrated in Scheme 2. The IR spectrum of compound **3** exhibited distinctive absorption bands at ν 3242 (NH), 2246, 2213 (2 C ≡ N), 1652 (C = O) and 1577 cm^− 1^ (C = C). The ^1^H NMR spectrum presented typical doublet and triplet signals at δ 3.17 and 5.47 ppm attributed to CH_2_-CN and H-5, respectively. Also, three singlet signals were recorded at δ 7.29, 7.53 and 8.34 ppm due to H-8, H-10 and H-4, respectively. A D_2_O-vanished signal due to NH proton was observed at δ 12.62 ppm. The ^13^C NMR spectrum of compound **3** showed characteristic signals at δ 70.4 (C-5), 108.2 (C-3), 112.8 (C-4a), 116.3 (C ≡ N), 117.2 (C ≡ N), 145.4 (C-4), 148.6 (C-6a), and 162.4 (C-2).

### Theoretical studies

#### Frontier molecular orbital energies and chemical reactivity

**Fig. 1:**
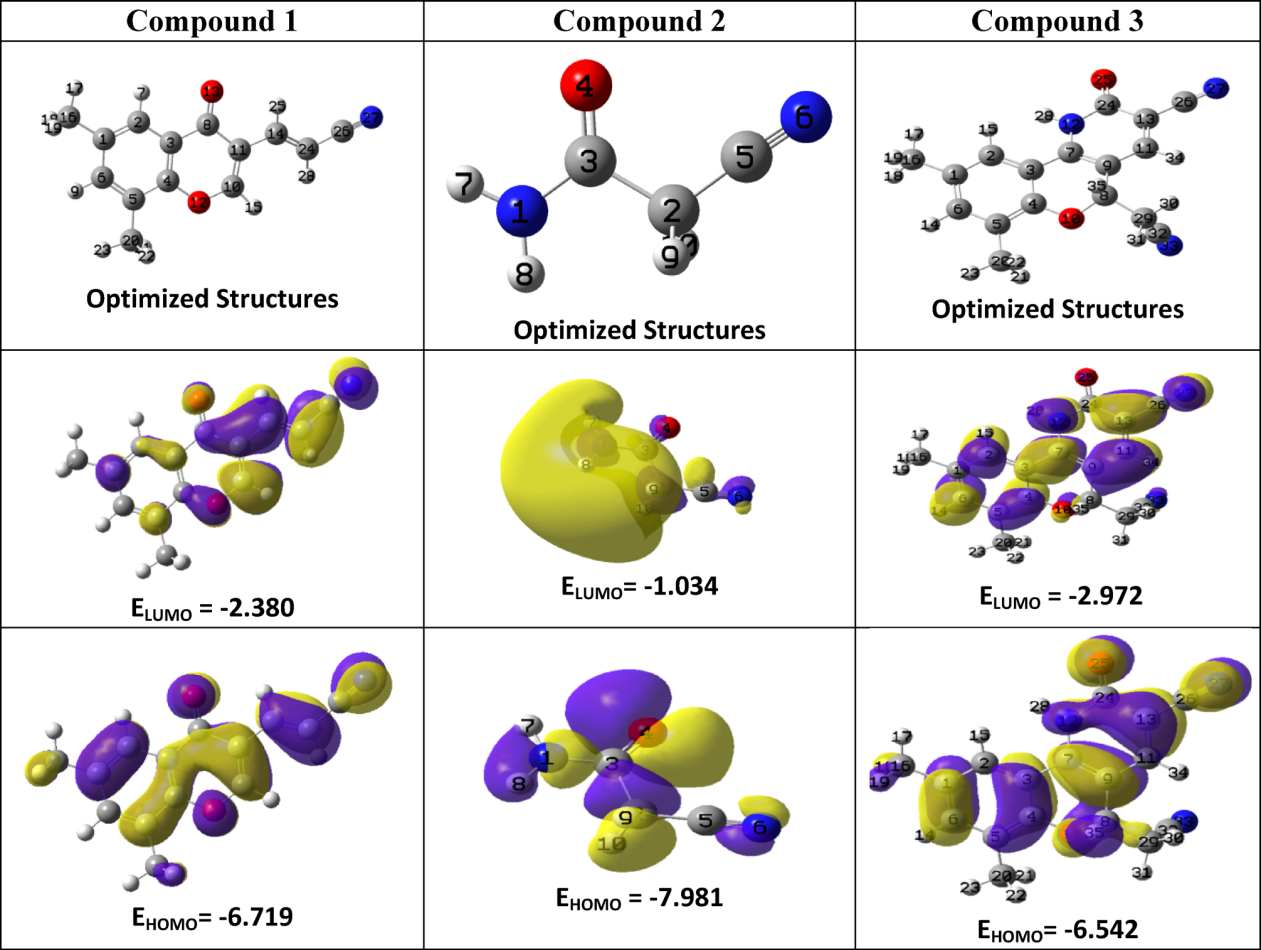
Molecular modeling and the electron density of HOMO and LUMO of compounds **1–3**.

Frontier molecular orbitals (FMOs) are fundamental in assessing a molecule’s optical, electronic, and reactive behavior, as well as its overall chemical stability^43^. The molecular structures of the investigated compounds were fully optimized using the DFT/B3LYP/6-311 + + G(d, p) method. Figure 1 illustrates the highest occupied molecular orbital (HOMO), the lowest unoccupied molecular orbital (LUMO), and the optimized geometries of the studied compounds^44^. The HOMO reflects the electron-donating capability of a molecule, while the LUMO represents its potential to accept electrons. Corresponding energy values (E_HOMO_ and E_LUMO_) are listed in Table 1. The energy gap (ΔE = E_LUMO_− E_HOMO_) serves as a critical indicator of chemical reactivity and kinetic stability; a larger ΔE denotes greater molecular stability. Among the evaluated compounds, compound **2** displayed the highest energy gap (ΔE = 6.947 eV), indicating superior chemical stability. In addition, several quantum chemical descriptors were computed in Table 1—including chemical potential (µ), electronegativity (χ), hardness (η), softness (S), electrophilicity index (ω), nucleophilicity (ε), and the maximum charge transfer capacity (ΔN_max_) using established theoretical formulas^45,46^.

6 $$I={\text{ }} - {E_{HOMO}}$$

7 $$Y={\text{ }} - {E_{LUMO}}$$

8 $$\:\:\chi\:=\:\frac{I+Y}{2}$$

9 $$\:\:\:\eta\:=\:\frac{I-Y}{2}$$

10 $$\:\:S=\:\frac{1}{\eta\:}$$

11 $$\:\:\:\omega\:=\frac{{\chi\:}^{2}}{2\eta\:}$$

12 $$\:\:\varepsilon\:=\:\frac{1}{\omega\:}$$

13 $$\:\mu\:\:\:=-\chi\:$$

14 $$\:\varDelta\:N=-\frac{\mu\:}{\eta\:}$$

Ionization potential (I, eV) represents the energy required to remove an electron from a molecule^46^, while electron affinity (Y, eV) quantifies the energy change when an electron is added. Chemical hardness (η, eV) reflects a molecule’s resistance to changes in its electron distribution, whereas softness (S, eV⁻¹) is its inverse^47^. Among the studied compounds, compound **2** has the highest hardness value (η = 3.473 eV), indicating that it is the least reactive and comparatively more rigid. In contrast, compound **3** shows the highest softness value (S = 0.560 eV^− 1^), making it the most reactive and chemically softer than the others. Additionally, electronegativity (χ, eV) describes a molecule’s tendency to attract electrons^48^, with compound **3** displaying the highest value (χ = 4.757 eV), suggesting strong electron-withdrawing characteristics. Chemical potential (µ, eV) measures the tendency of electrons to escape from a system; the negative values observed for the studied compounds indicate overall molecular stability.

Moreover, Parr et al.^49^ introduced the electrophilicity index (ω) as a parameter to quantify the decrease in energy when a molecule gains an additional electronic charge (ΔN_max_) during interactions between electron donors and acceptors. Compound **3** exhibits a high ω value (6.338 eV) along with a significant ΔN_max_ (2.665), indicating its strong electrophilic nature. Conversely, nucleophilicity (ε, eV^− 1^) measures a molecule’s tendency to donate electrons^50^, where compound **2** showing the highest ε value (0.342 eV^− 1^), making it the most potent nucleophile.

**Table 1:** Global reactivity descriptors of the studies compounds **1–3**.

Parameters	Compound 1	Compound 2	Product 3 (CCPC)
E_HOMO_	-6.719	-7.981	-6.542
E_LUMO_	-2.380	-1.034	-2.972
IP (eV)	6.719	7.981	6.542
EA (eV)	2.380	1.034	2.972
ΔE (eV)	4.339	6.947	3.570
η (eV)	2.169	3.473	1.785
µ (eV)	-4.549	-4.508	-4.757
ω (eV)	4.771	2.925	6.338
ε (eV^− 1^)	0.209	0.342	0.158
S (eV^− 1^)	0.461	0.288	0.560
χ	4.549	4.508	4.757
∆N_max_	2.097	1.298	2.665

#### Molecular electrostatic potential (MEP)

**Fig. 2:**
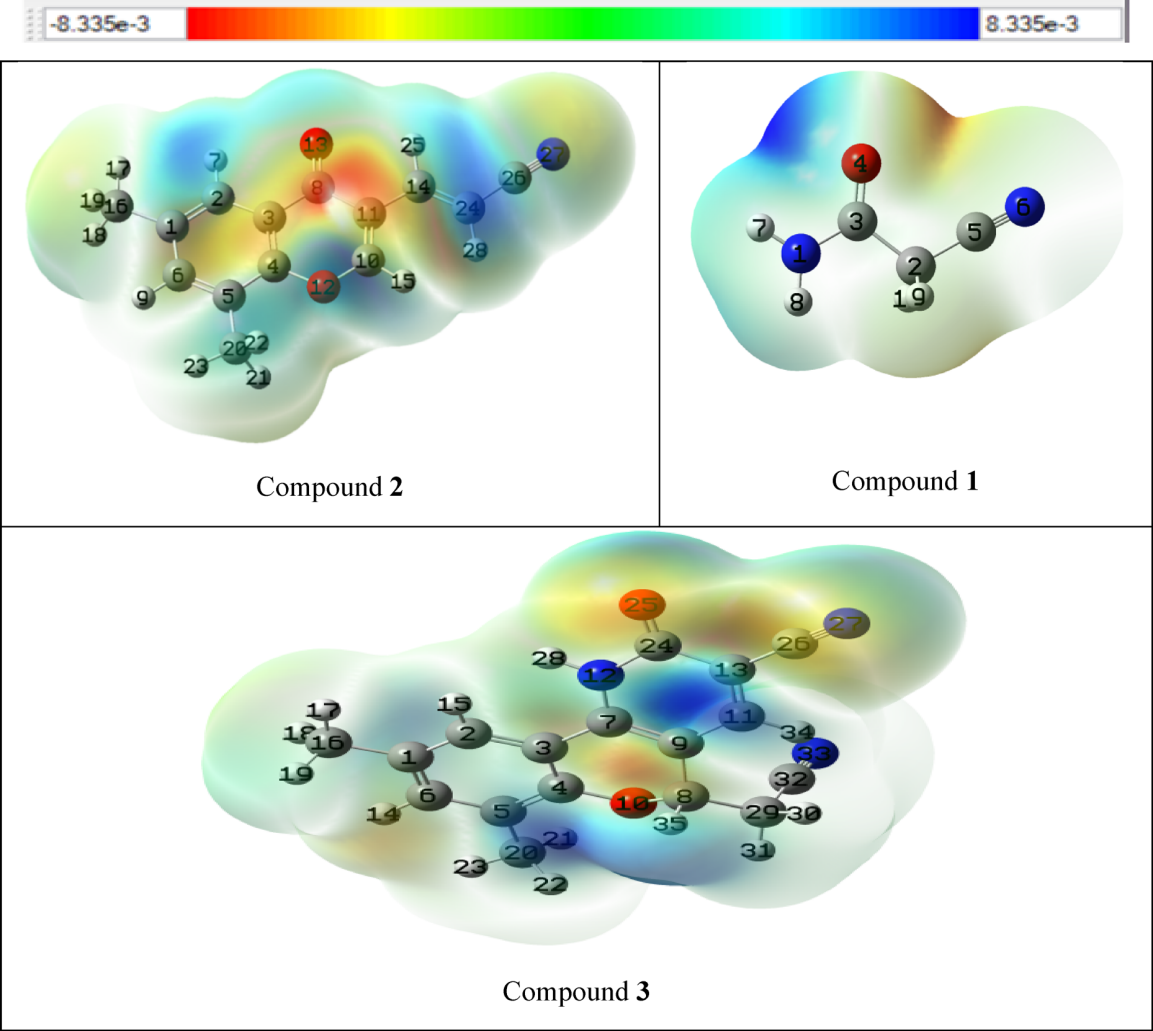
Molecular electrostatic potential of compounds **1–3**.

Molecular electrostatic potential (MEP) mapping offers insights into a molecule’s size, hydrogen bonding interactions, and overall charge distribution resulting from electrons and nuclei. It helps predict sites of electrophilic attack (electron-rich regions with high electronegativity) and nucleophilic attack (electron-deficient regions with high positive electrostatic potential)^51,52^. The electrostatic potential of the molecules was calculated using DFT at the B3LYP/6-311 + + G (d, p) level^53^. Figure 2 illustrates the MEP maps of the studied compounds.

The MEP map represents different electrostatic potentials through a color gradient: blue > green > yellow > orange > red. Blue areas indicate low electron density, while red regions represent high electron density^54^. The intense blue areas, primarily located on hydrogen and carbon atoms, signify the main electrophilic sites, suggesting a strong tendency to attract with nucleophilic reagents. Conversely, regions depicted in deep yellow and red colors mainly associated with nitrogen and oxygen atoms, indicating nucleophilic sites, suggesting a strong tendency to interact with electrophilic reagents. The presence of lone electron pairs as well as high electronegativity of these atoms may be the reasons for creating regions of negative electrostatic potential, affirming their nucleophilic character^55^.

Herein, the MEP map for starting compound **1** supported the mechanism depicted in Scheme 1, where C-2 position has deep blue color indicating high suitability for nucleophilic attack by the reagent **2**. The blue color at C-2 position was furnished due to low electron density (electrophilic site) achieved by electron withdrawing mesomeric effects of both C = O and C ≡ N, as well as the inductive effect of oxygen atom at position 1. In addition, the MEP map of the product **3** revealed deep blue color over C-4, and this mean low electron density (electrophilic nature) which achieved by electron withdrawing effect of both C = O and C ≡ N functions. Therefore, this map supports the ^1^H NMR results which displayed H-4_pyridine_ at high chemical shift (δ) value as compared with other aromatic protons (see Table 3). Since, high δ value means low magnetic field (deshielding) due to low electron density (electrophilic nature in MEP map and therefore blue color, Fig. 2). On the other hand, a yellow color was seen at C-4a in the MEP of compound **3**, and this may attribute to nucleophilic nature of this position due to electron repelling effect of NH group which increased the electron density over this site. Hence, high magnetic field (shielding) over C-4a position which leads to low chemical shift (δ) value in the ^13^C NMR spectra as compared with other aromatic carbons (see Table 3; Fig. 2).

#### FT- IR Vibrational Analysis

The IR Vibrational Analysis is of considerable importance, as it enables accurate identification of molecules based on their distinctive infrared absorption profiles, thereby allowing precise determination of a substance’s chemical composition^56^. DFT calculations of vibrational frequencies have shown excellent agreement with the vibrational modes of organic compounds^56^. For the current study, the theoretical frequencies were computed using the B3LYP/6-311 + + G(d, p) level of theory and corrected using a scaling factor of 0.961 to minimize systematic errors. Table 2 present both the theoretical and experimental infrared vibrational frequencies for compound **3**. The experimental and calculated FT-IR spectra are illustrated in Fig. S1.

In the IR spectra of compound **3**, the NH stretching vibration was recorded experimentally at ν 3242 cm^− 1^, which was found theoretically at ν 3255 cm^− 1^. The experimental IR spectrum showed the absorption bands of the two C ≡ N groups at ν 2246 and 2213 cm^− 1^, while the theoretical values were observed at ν 2250 and 2220 cm^− 1^. Moreover, the measured stretching motion for C = O group was seen at ν 1652 cm^− 1^, which matched with the theoretical value at ν 1670 cm^− 1^.

**Fig. 3:**
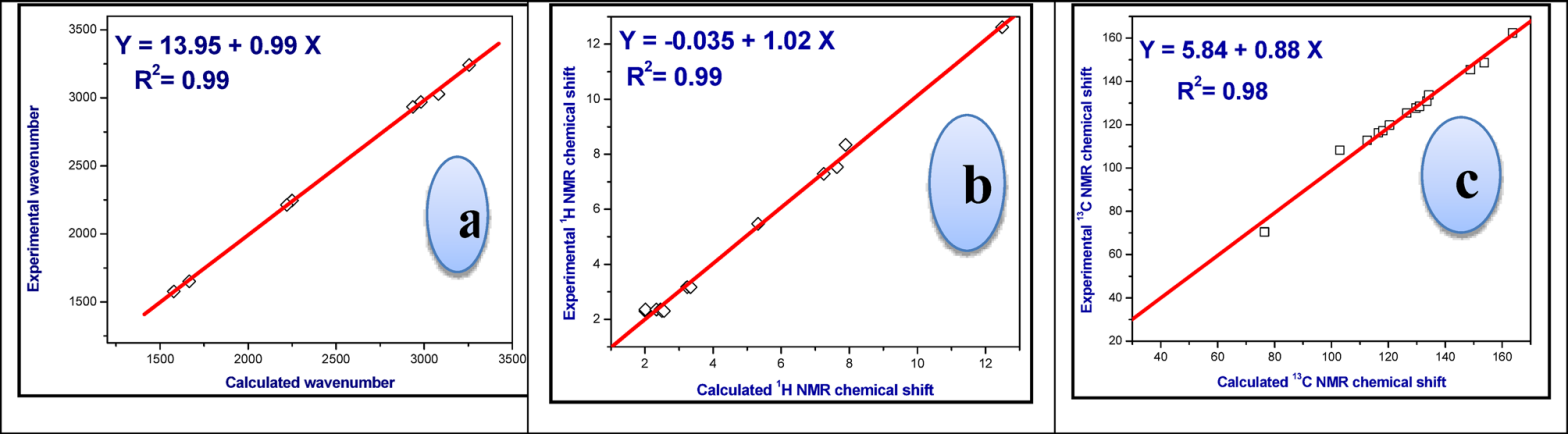
Plots of the relationships of the calculated *versus* experimental **(a)** IR wavenumbers, **(b)** ^1^H NMR and **(c)** ^13^C NMR chemical shifts of **CCPC**, **3**.

Figure 3a showed a relationship between the wavenumbers derived from experimental observations and those computed theoretically for the functional groups; and exhibit a correlation coefficient (R²) of 0.99.

**Table 2:** Experimental and theoretical frequencies and corresponding vibrational assignments of **CCPC** (**3**) at the B3LYP/6-311 + + G (d, p).

Compound 3 (CCPC)		
υ_exp_.(cm^− 1^)	υ_the_.(cm^− 1^)	Assignment
3242	3255	NH
3027	3081	CH_aromatic_
2971, 2934	2981, 2936	CH_aliphatic_
2246, 2213	2250, 2220	2 C ≡ N
1652	1670	C = O
1577	1578	C = C

#### ^1^H NMR and ^13^C NMR spectroscopy

An effective strategy for investigating the structures of organic molecules involves integrating quantum computational chemistry methods with nuclear magnetic resonance (NMR) spectroscopy. The GIAO approach, combined with the B3LYP/6-311 + + G basis set, was employed to theoretically compute the ¹H and ¹³C NMR chemical shifts of the synthesized compounds^57,58^. Table 3 presents a comparison between the calculated and experimental ¹H NMR chemical shifts, while Fig. S2 shows the ¹H NMR spectra recorded in DMSO.

**Table 3:** Calculated and experimental ^1^H and ^13^C NMR chemical shifts of **CCPC** on B3LYP/6-311 + + G(d, p) basis set.

^1^H NMR			^13^C NMR		
**Atoms**	Calculated	Experimental	Atoms	Calculated	Experimental
**17-H**	2.008296	2.30	**20-C**	17.25262	17.5
**23-H**	2.012475	2.36	**16-C**	21.01514	20.4
**21-H**	2.336835	2.36	**29-C**	21.28556	22.3
**22-H**	2.45021	2.36	**8-C**	76.4621	70.4
**18-H**	2.495781	2.30	**13-C**	102.9234	108.2
**19-H**	2.55246	2.30	**9-C**	112.5739	112.8
**30-H**	3.236307	3.17	**26-C**	116.5117	116.3
**31-H**	3.333138	3.17	**32-C**	118.0458	117.2
**35-H**	5.319709	5.47	**3-C**	120.4037	119.8
**14-H**	7.247692	7.29	**7-C**	126.4159	125.4
**15-H**	7.639213	7.53	**1-C**	129.6899	127.7
**34-H**	7.887824	8.34	**5-C**	130.9913	128.5
**28-H**	12.48683	12.62	**6-C**	133.5516	130.9
			**2-C**	134.2141	133.7
			**11-C**	148.8349	145.4
			**4-C**	153.7202	148.6
			**24-C**	163.6628	162.4

In the ^1^H NMR spectra of compound **3**, It was noted that, the three protons for each methyl group was recorded in the experimental chart as one signal, whereas three signals were recorded in the computed chart; and this may assign to different electron densities around each proton and hence different magnetic field (Table 3). Also, the two protons of CH_2_CN group were recorded experimentally at δ 3.17 ppm, whereas theoretically calculated as two signals at δ 3.23 and 3.33 ppm; and this may attribute to different orientation in space and consequently different chemical environments. The chemical shifts of the two benzo protons were seen experimentally at 7.29 and 7.53 ppm, whereas the calculated values were 7.25 and 7.64 ppm, respectively. The chemical shifts of H-5 and H-8 protons were observed experimentally at δ 5.47 and 8.34 ppm, while the computed values were at δ 5.32 and 7.89 ppm. The experimental chemical shift of the NH proton was found at δ 12.62 ppm, while the computed signal was determined at δ 12.48 ppm.

The ^13^C NMR spectra of compound **3** are displayed in Fig. S3. Table 3 shows the data of theoretical and experimental ^13^C-NMR chemical shifts. For example, the theoretical/experimental δ value of C = O was found at δ 163.6/ 162.4 ppm. In addition, the theoretical δ values of the two C ≡ N functions were found at δ 116.5 and 118.0 ppm, which closely agree with that experimentally recorded at δ 116.3 and 117.2 ppm. The experimental values for C-5 and C-4 were seen at δ 70.4 and 145.4 ppm, while the calculated values were δ 76.5 and 148.8 ppm. Moreover, the carbon atoms of the two CH_3_ groups were computed at δ 17.2 and 21.0 ppm which closed to the observed signals at δ 17.5 and 20.4 ppm. The relatively high chemical shift (δ) of C-5 as compared with other sp3 hybridized carbon may attribute to the deshielding achieved by the adjacent high electronegative oxygen atom.

The experimental ^1^H and ^13^C NMR chemical shifts were plotted with the theoretical chemical shifts and the correlation coefficients (R^2^) are 0.99 and 0.98, respectively, as shown in Figs. 3b, c. Thus, the computed values are in good agreement with experimental values.

#### Drug similarity and in-silico ADME anticipation

Swiss ADME was utilized to carry out computational ADME analysis on the synthesized compounds to evaluate their physicochemical properties and drug-likeness characteristics^59,60^. All compounds comply with Lipinski’s rule of five, which includes the following criteria: MlogP less than 5, no more than 5 hydrogen bond donors (HBD), no more than 10 hydrogen bond acceptors (HBA), and a molecular weight (MW) under 500 amu. The molecular weights of the current compounds range from 84 to 291 g/mol, which are within the acceptable range (Table 4). The HBA values fall between 2 and 4, while HBD values between 0 and 1; hence both within the permissible limits. Furthermore, the predicted MlogP values range from − 1.36 to 1.21 (Table 4).

In addition to Lipinski’s criteria, Veber’s rule was also applied to assess drug-likeness. According to this rule: (a) the total polar surface area (TPSA), which is related to bioavailability, should not exceed 140 Å². The TPSA values for the studied compounds range from 54 to 89.67 Å². (b) The number of rotatable bonds should be fewer than 10, and all synthesized compounds meet this requirement (see Table 4). Collectively, the data indicate that all compounds possess favourable properties for oral bioavailability, as they satisfy both Lipinski’s and Veber’s guidelines.

**Table 4:** Lipinskiʼs and Veber’s rules for drug-likeness of compounds **1**–**3**.

Compound	HBA	HBD	MW	Log *P*	TPSA	Rotatable bond	Lipinski #violations	Veber violations
**1**	3	0	225.24	1.21	54.00	1	0	0
**2**	2	1	84.08	-1.36	66.88	1	0	0
**3**	4	1	291.30	1.06	89.67	1	0	0

#### **Non-linear optical** (**NLO) properties**

**Fig. 4:**
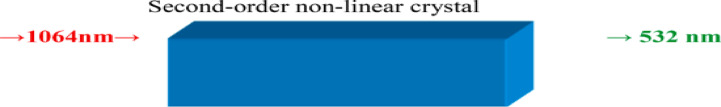
The second harmonic generation.

Nonlinear optical (NLO) properties refer to the ability of a material to interact with high-intensity light, such as that from a laser, and convert light of longer wavelengths into light of shorter wavelengths. One of the key NLO phenomena is second harmonic generation (SHG), in which incident light is effectively transformed so that its wavelength is halved (Fig. 4). This occurs when the material absorbs the incoming light and, through a nonlinear optical process, emits light at twice the frequency (i.e., half the wavelength) of the original beam.

Single crystals of nonlinear optical (NLO) materials have found widespread applications in various advanced technologies, including semiconductors, infrared detectors, solid-state lasers, photosensitive devices, and crystalline thin films used in microelectronics^61,62^. To explore the correlation between the electronic structure and NLO properties of the studied compound, theoretical calculations were performed using the B3LYP/6-311 + + G(d, p) level of theory in the gas phase and in different solvents (water, ethanol, acetone, and DMSO). Key NLO parameters including the total static dipole moment (µ), mean polarizability (⟨α⟩), polarizability anisotropy (Δα), mean first-order hyperpolarizability (β), hyper-Rayleigh scattering coefficient (*β*_*HRS*_), and depolarization ratio (DR) are summarized in Table 5.

In this study, *p*-nitroaniline (PNA) was employed as a standard reference molecule for evaluating nonlinear optical (NLO) properties, due to the lack of experimental NLO data for the investigated compound. The values of the first-order hyperpolarizability (β), presented in Table 5, indicate that the studied compound (**CCPC**) exhibits β values approximately 3.5 times higher than those of PNA across all media. Notably, the β values in water and ethanol are significantly higher compared to those in the gas phase, acetone, and DMSO. For comparison, the reported first-order hyperpolarizability of PNA is 15.5 × 10⁻³⁰ esu, as documented by T. Gnanasambandan et al.^63^. The analysis further reveals that **CCPC** demonstrates a ~ 2.5-fold enhancement in β values over PNA in all solvents, with water and ethanol again showing the highest values. Additionally, the relatively lower β values observed in some environments suggest potential for increased optical selectivity. Overall, these findings suggest that the **CCPC** compound possesses promising nonlinear optical properties and may serve as a potential candidate for optoelectronic applications.

**Table 5:** Total static dipole moment (µ), the mean polarizability (˂α˃), the anisotropy of the polarizability (Δα), and the mean first-order hyperpolarizability (˂β˃), for the studied compound **CCPC** in gas phase, water, ethanol, acetone, and DMSO computed at B3LYP/6-311 + + G(d, p).

Medium	Property	CCPC
Gas phase	***µ***,*** D*** *ebye* ^*a*^	7.98
water		12.22
ethanol		11.87
acetone		11.31
DMSO		11.62
Gas phase	$<\alpha> \times 10^{-24} esu^b$	25.65
water		36.55
ethanol		31.58
acetone		33.98
DMSO		34.28
Gas phase	$\Delta\alpha \times 10^{-24} esu$	63.62
water		64.65
ethanol		64.87
acetone		64.27
DMSO		64.29
Gas phase	$˂ \alpha ˃ \times 10^{-30} esu^c$	85.53
water		128.74
ethanol		126.32
acetone		120.78
DMSO		122.41

^a, b, c^ PNA results (2.44, 22, 15.5) are taken from references^61–63^.

#### NBO analysis

Natural Bond Orbital (NBO) analysis was originally developed as a theoretical framework to investigate hybridization patterns and covalency within polyatomic wave functions, particularly in systems exhibiting hydrogen bonding and other strongly bound van der Waals interactions ^64^. In this formalism, covalent interactions within molecules are represented by the filled NBOs (σ) that constitute the ‘‘natural Lewis structure’’^65^. In contrast, noncovalent interactions are elucidated through the transformation of canonical molecular orbitals into NBOs, which also generate formally unoccupied orbitals absent in the idealized Lewis structure. Within this notation, σ and σ* are employed in a generalized manner to denote occupied and unoccupied orbitals, respectively. The occupied orbitals may correspond to core orbitals (CR), lone pairs (LP), or bonding orbitals (σ, π), whereas the unoccupied orbitals can represent antibonding orbitals (σ*, π*) or higher-energy Rydberg-type orbitals (RY*).

Also, NBO analysis provides insight into intra- and intermolecular interactions by identifying electron delocalization between donor and acceptor orbitals. These interactions occur specifically between filled orbitals of Lewis-type (bonding or lone pairs) and corresponding empty non-Lewis orbitals (antibonding or Rydberg). The stabilization energies arising from such donor–acceptor interactions are quantitatively evaluated using second-order perturbation theory ^64^. The stabilization energy, E^(2)^, which reflects the degree of electron delocalization from a donor orbital (*i*) to an acceptor orbital (*j*), is determined using the following expression ^65^.

15 $${E^{(2)}}={\text{ }}\Delta Eij{\text{ }}=qi(F(ij){\text{ }}2{\text{ }}/\varepsilon j-\varepsilon i),$$

where Fijis (*ij*) denotes the off-diagonal element of the NBO Fock matrix, *qi* ​ represents the occupancy of the donor orbital, and and correspond to the diagonal energies of the donor and acceptor NBOs, respectively.

This approach is highly effective for identifying charge delocalization pathways, such as transitions or conjugative interactions, within different regions of a molecular framework. The extent of hyper-conjugative interactions between donor and acceptor orbitals is directly correlated with the associated stabilization energy. In this context, the second-order perturbation stabilization energies provide a quantitative measure of conjugation throughout the system. For the current compound **CCPC**, nucleophilic hyper-conjugative interactions were found to generate stabilization energies of 112.7, 85.59, 58.81, 57.91, and 55.58 kJ·mol⁻¹ across all studied solvents. Notably, the stabilization energies in polar solvents (water and ethanol) are higher compared with those in less polar media (gas phase, acetone, and DMSO), reflecting enhanced charge delocalization in the former. These values correspond to transitions of the following types: LP(1)(O10)→π*(C4–C8), LP(1)(N27)→π*(C7–O25), LP(1)(N12)→π*(C24–O25), LP(1)(O25)→π* (C13–C24), and LP(1)(O25)→π* (C2–C6) (Table 6).

The electron delocalization process exhibits significant stabilization energy, with values up to 85.6 kJ·mol⁻¹, which can be attributed to resonance effects within the molecule. Remarkably, the investigated compound **CCPC** displays comparatively higher stabilization energies, highlighting the enhanced stability of its electronic molecular framework.

As a result, The NBO analysis confirmed significant donor–acceptor interactions and pronounced electron delocalization in compound **CCPC**. The relatively high stabilization energies, particularly in polar solvents, reflect strong hyper-conjugative and resonance effects that enhance the electronic stability of the molecular framework. These findings provide clear evidence of the conjugative nature of the system and its solvent-dependent stabilization behavior.

**Table 6:** Second Order Perturbation Interaction Energy Values Computed in the NBO Basis for the studied compound **CCPC** in gas phase, water, ethanol, acetone, and DMSO computed at B3LYP/6-311 + + G(d, p).

Donor	Acceptor	E^(2)a^(kcal/mol)					NBO	Population				
		Gas phase	water	ethanol	acetone	DMSO		Gas phase	water	ethanol	acetone	DMSO
π C2-C6	π* C1-O10	48.61	76.21	72.91	65.39	65.39	C2-C6	1.87	1.78	1.79	1.82	1.82
LP (1) O10	π C4-C8	112.7	75.21	71.91	64.37	64.38	C4-C8	1.85	1.77	1.78	1.80	1.80
LP(1) N12	π* C24-O25	58.81	70.3	68.7	62.52	62.28	LP(1) N12	1.70	1.68	1.68	1.68	1.68
LP (1) O25	π* C13-C24	57.91	69.3	67.7	61.52	61.28	LP(1) N27	1.68	1.67	1.67	1.67	1.67
LP (1) N27	π* C7-O25	85.59	107.1	105.1	106.0	107.2	LP (1)O10	1.10	1.99	1.99	1.99	1.99
LP (1) N33	RY*C1	23.35	22.81	23.00	23.29	23.30	LP (2) O25	1.90	1.92	1.92	1.91	1.91
LP (2) O10	σ*C1-C2	24.63	20.10	20.71	22.11	22.11	LP (1) N33	1.88	1.80	1.79	1.81	1.80
LP (2) N27	σ* C1-N27	45.55	37.10	38.41	41.44	41.55	C1-O10	0.27	0.37	0.35	0.33	0.33
LP (1) O25	π* C2-C6	55.58	90.51	89.41	83.03	84.54	C2-C6	0.27	0.34	0.33	0.33	0.33
LP (2) N33	π* N12-H28	139.4	133.8	132.5	134.8	135.1	C4-C8	0.37	0.41	0.40	0.42	0.42
π* C2-C6	π* C1-O10	116.6	110.1	111.2	307.7	302.9						

#### Potential energy diagram and intrinsic reaction coordinates (IRC)

**Fig. 5:**
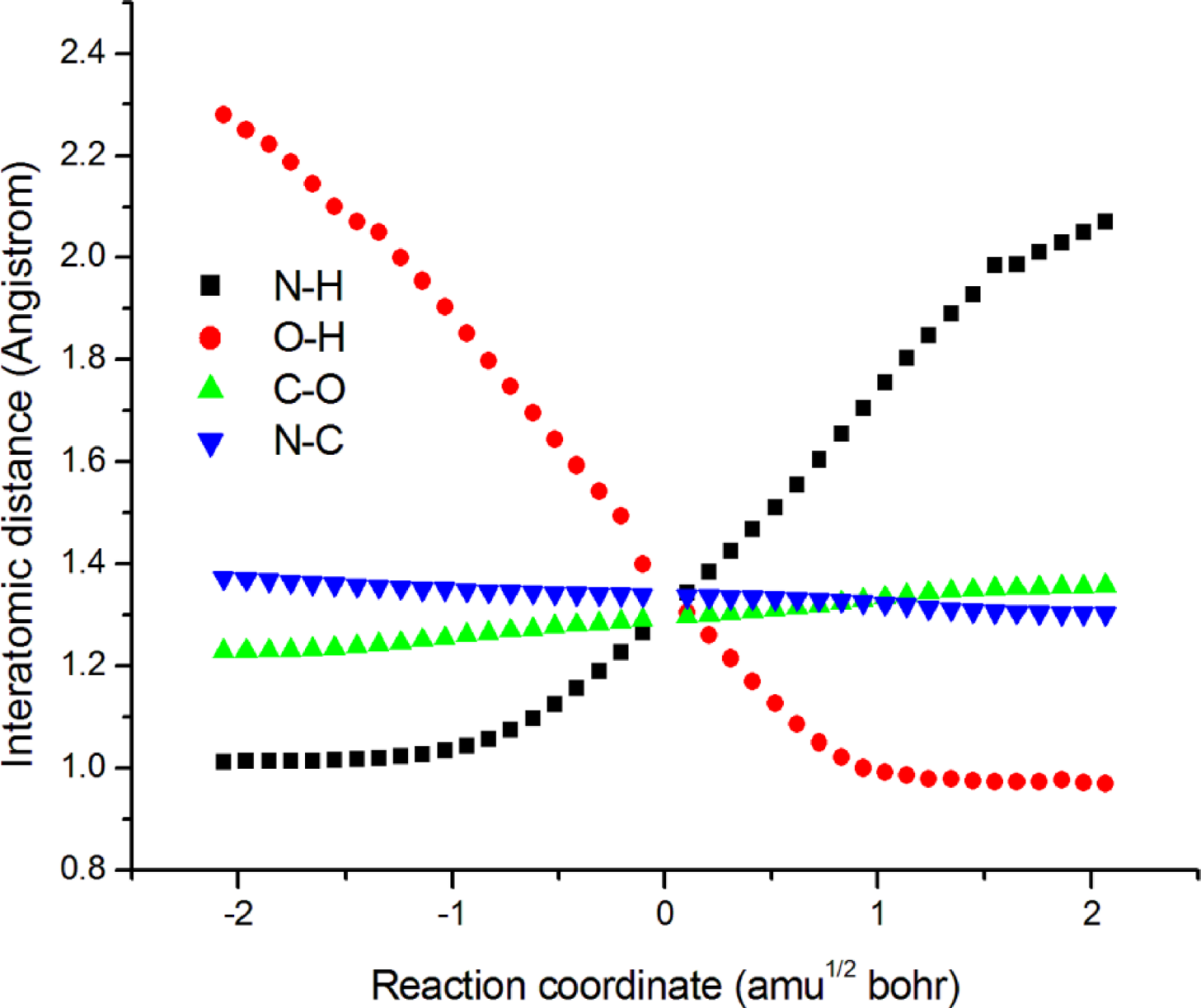
Change of bond lengths (angstroms) along reaction coordinates for **CCPC** at B3LYP/6-311 + + G(d, p) level.

**Fig. 6:**
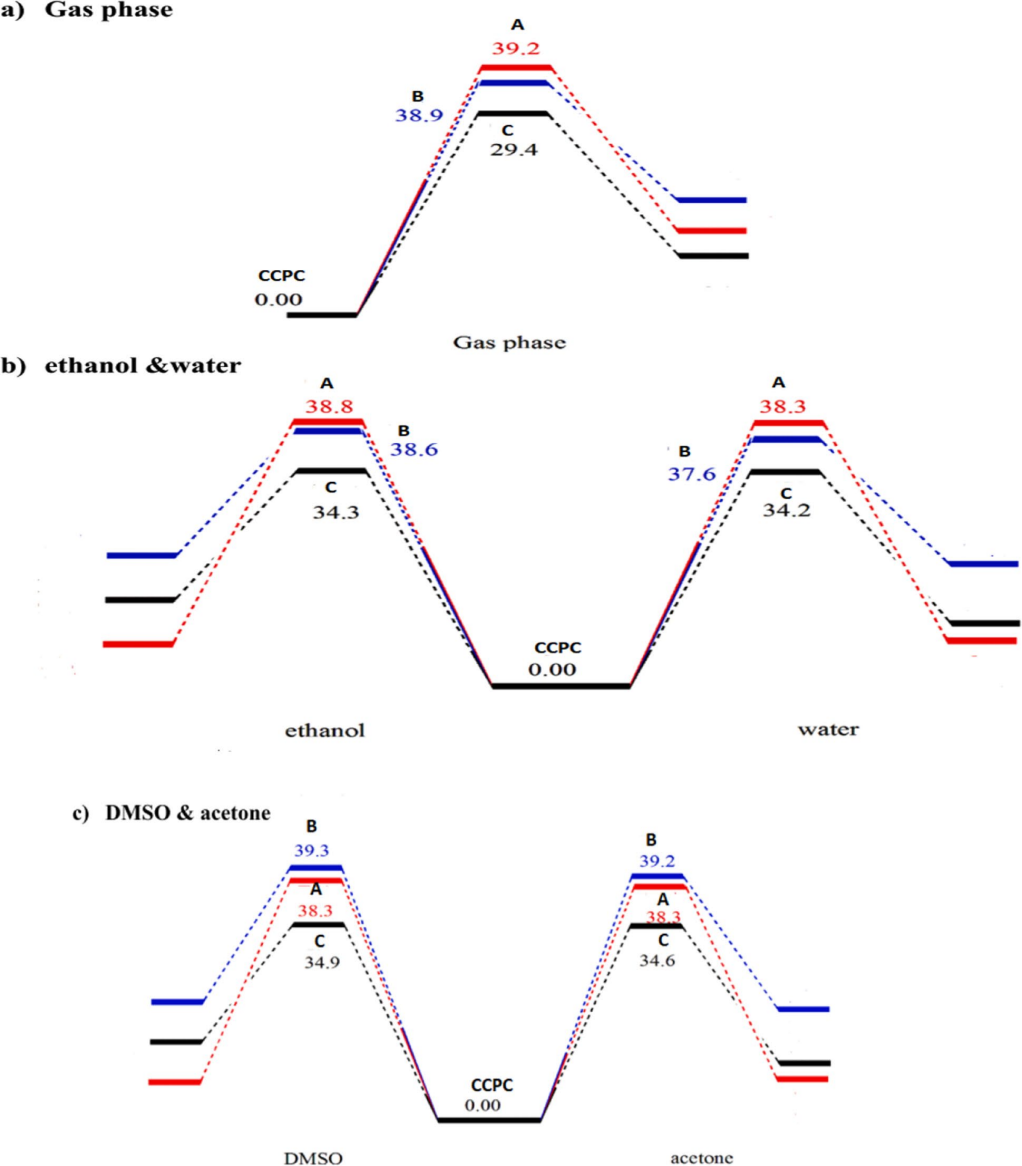
Potential energy diagram $\varDelta$*(E*_*0K*_, $\varDelta$ _*0K*_^*†*^, in kcal/mol) for different conversions of **CCPC** in different solvents using B3LYP/6-311 + + G(d, p) level *(T = 0 K and P  = 1 atm)*.

As illustrated in Scheme 1, the suggested mechanism of **CCPC** can proceed *via* an intramolecular hydrogen atom transfer (HAT), indicative of a complex conversion pathway. The variations in bond lengths during this process for intermediates **A**, **B**, and **C** along the intrinsic reaction coordinate (IRC) in the gas phase are presented in Fig. 5. Correspondingly, Fig. 6 displays the potential energy profile of the **CCPC** decomposition calculated at the B3LYP/6-311 + + G (d, p) level. Barrier heights and reaction energies derived from this pathway are summarized in Table 7. Analysis of Figs. 5 and 6 reveals that, along the IRC pathway, the O–H bond gradually forms in tandem with the cleavage of the N–H bond, with both bond evolution curves intersecting at s = 0 amu^1/2^ bohr. Additionally, formation of the N–C bond and cleavage of the C–O bond occur smoothly throughout the reaction coordinate, indicating a well-coordinated intramolecular rearrangement.

According to the data in Table 7, structure **A** exhibits pronounced imaginary vibrational frequencies in the range of 1869.7 *i* to 1920.1 *i*, as calculated using the B3LYP/6-311 + + G(d, p) method. These high-magnitude imaginary frequencies suggest a significant contribution from quantum mechanical tunneling in this reaction pathway. Analysis of the corresponding energy profile indicates that although the pathway involving structure **A** is kinetically the least favorable, it is thermodynamically the most preferred—being the least endothermic route for the decomposition of **CCPC**. The computed activation barrier heights (along with reaction energies) are 39.2, 38.3, 38.8, 38.3, and 38.3 kcal/mol in the gas phase, water, ethanol, acetone, and DMSO, respectively, at the B3LYP/6-311 + + G(d, p) level of theory.

Among the investigated pathways for **CCPC** decomposition, the formation of structure **B** corresponds to the most endothermic route on the potential energy surface across all studied media. From a kinetic standpoint, intermediate **B** represents the second most favorable pathway in the gas phase, water, and ethanol; however, it becomes the least favorable route in acetone and DMSO. The calculated activation barrier heights (and reaction energies) for this pathway are 38.9, 37.6, 38.6, 39.2, and 39.3 kcal/mol in the gas phase, water, ethanol, acetone, and DMSO, respectively, as shown in Table 7, using the B3LYP/6-311 + + G(d, p) level of theory.

In contrast, the formation of structure **C** is associated with prominent imaginary frequencies of 1622.34 *i*, 1699.72 *i*, 1689.29 *i*, 1682.52 *i*, and 1685.92 *i* in the gas phase, water, ethanol, acetone, and DMSO, respectively (Table 7). This pathway is identified as the most kinetically favorable route for **CCPC** decomposition. The corresponding activation energies (and reaction enthalpies) for this route are 29.4, 34.2, 34.3, 34.6, and 34.9 kcal/mol in the gas phase, water, ethanol, acetone, and DMSO, respectively, calculated at the same level of theory.

**Table 7:** The activated energies ^a^ (in kcal mol^−1^) and the relative energies^a^ for studied pathways in different solvents using the B3LYP/6-311 + + G(d, p) methods. (*P* = 1 bar, *T* = 298 K)

Medium	Species	B3LYP/6-311 + + G(d, p)			
		Imiginary freqency(cm^−1^)	Δ*E*_0K_^†^	Δ*H*_298K_^†^	Δ*G*_298K_^†^
Gas phase	**A**	-1869.70	39.24	39.14	39.33
water		-1920.14	38.28	38.12	38.40
ethanol		-1910.16	38.81	38.70	38.85
acetone		-1891.52	38.28	38.22	38.20
DMSO		-1885.35	38.32	38.23	38.28
Gas phase	**B**	-1606.95	38.87	38.78	38.94
water		-1684.14	37.58	37.38	37.82
ethanol		-1674.26	38.56	38.44	38.70
acetone		-1660.44	39.17	39.10	39.25
DMSO		-1662.59	39.24	39.14	39.35
Gas phase	**C**	-1622.34	29.43	29.33	29.54
water		-1699.72	34.20	34.12	34.19
ethanol		-1689.29	34.29	34.42	33.31
acetone		-1682.52	34.61	34.66	34.35
DMSO		-1685.92	34.91	35.00	34.49
			**Δ** ***E***_**0 K**_	**Δ** ***H***_**298 K**_	**Δ** ***G***_**298 K**_
Gas phase	**CCPC**		10.14	10.13	10.21
water			6.59	6.73	6.18
ethanol			7.37	7.55	6.92
acetone			7.60	7.76	7.29
DMSO			7.70	7.83	7.47

^a^ energies calculated relative to the parent molecule.

#### Chemical kinetics

The rate constants for all unimolecular hydrogen atom transfer (HAT) reactions involved in the formation of **CCPC** (Scheme 1) were computed using transition state theory (TST) in conjunction with the Eckart tunneling correction. These calculations were carried out under standard conditions (*P* = 1 atm) across a temperature range of 250–400 K. The results obtained from both methods—TST alone and TST with tunneling correction—were found to be closely aligned and comparable in magnitude. A summary of the forward and reverse rate constants for the various **CCPC** transformation pathways, incorporating Eckart tunneling corrections, is presented in Table 8 for the specified temperature range.

**Table 8:** Eckart tunneling correction for forward and reverse reactions for different transformation of **CCPC** at B3LYP/6-311 + + G(d, p) level (*P* = 760 torr).

*T*(*K*)	Medium	Forward reaction			Reverse reaction		
		k_(R→A)_	k_(R→B)_	k_(R→C)_	k_(CCPC→A)_	k_(CCPC→B)_	k_(CCPC→C)_
250	Gas phase	8.91E + 05	2.54E + 03	6.30E + 03	9.20E + 05	2.79E + 03	5.21E + 03
	water	3.45E + 06	1.29E + 04	2.15E + 04	3.71E + 06	1.40E + 04	2.28E + 04
	ethanol	2.84E + 06	9.53E + 03	1.66E + 04	3.04E + 06	1.04E + 04	1.72E + 04
	acetone	1.72E + 06	6.63E + 03	1.43E + 04	1.80E + 06	7.23E + 03	1.50E + 04
	DMSO	1.56E + 06	2.92E + 04	1.50E + 04	1.61E + 06	7.51E + 03	1.54E + 04
275	Gas phase	2.73E + 04	3.01E + 02	5.33E + 02	2.76E + 04	3.18E + 02	4.73E + 02
	water	8.11E + 04	9.92E + 02	1.41E + 03	8.47E + 04	1.05E + 03	1.47E + 03
	ethanol	6.90E + 04	8.00E + 02	1.17E + 03	7.33E + 04	8.46E + 02	1.19E + 03
	acetone	4.60E + 04	6.17E + 02	1.05E + 03	4.78E + 04	6.51E + 02	1.08E + 03
	DMSO	4.15E + 04	1.57E + 03	1.09E + 03	4.33E + 04	6.71E + 02	1.11E + 03
298	Gas phase	2.47E + 03	7.76E + 01	1.12E + 02	2.48E + 03	8.01E + 01	1.04E + 02
	water	5.86E + 03	1.86E + 02	2.39E + 02	6.04E + 03	1.93E + 02	2.45E + 02
	ethanol	5.17E + 03	1.60E + 02	2.08E + 02	5.34E + 03	1.66E + 02	2.11E + 02
	acetone	3.69E + 03	1.33E + 02	1.91E + 02	3.78E + 03	1.37E + 02	1.95E + 02
	DMSO	3.41E + 03	2.36E + 02	1.98E + 02	3.49E + 03	1.40E + 02	2.00E + 02
325	Gas phase	3.15E + 02	2.64E + 01	3.30E + 01	3.17E + 02	2.69E + 01	3.17E + 01
	water	6.03E + 02	4.86E + 01	5.73E + 01	6.16E + 02	4.96E + 01	5.81E + 01
	ethanol	5.44E + 02	4.40E + 01	5.21E + 01	5.57E + 02	4.48E + 01	5.24E + 01
	acetone	4.24E + 02	3.87E + 01	4.91E + 01	4.29E + 02	3.94E + 01	4.96E + 01
	DMSO	3.97E + 02	5.32E + 01	5.03E + 01	4.03E + 02	4.01E + 01	5.06E + 01
350	Gas phase	8.34E + 01	1.35E + 01	1.56E + 01	8.35E + 01	1.37E + 01	1.53E + 01
	water	1.35E + 02	2.11E + 01	2.36E + 01	1.37E + 02	2.13E + 01	2.38E + 01
	ethanol	1.25E + 02	1.96E + 01	2.21E + 01	1.26E + 02	1.99E + 01	2.22E + 01
	acetone	1.03E + 02	1.79E + 01	2.12E + 01	1.04E + 02	1.81E + 01	2.13E + 01
	DMSO	9.84E + 01	2.16E + 01	2.15E + 01	9.93E + 01	1.83E + 01	2.16E + 01
375	Gas phase	3.28E + 01	8.42E + 00	9.32E + 00	3.28E + 01	8.47E + 00	9.19E + 00
	water	4.68E + 01	1.18E + 01	1.28E + 01	4.72E + 01	1.18E + 01	1.29E + 01
	ethanol	4.40E + 01	1.12E + 01	1.22E + 01	4.44E + 01	1.13E + 01	1.22E + 01
	acetone	3.83E + 01	1.04E + 01	1.18E + 01	3.85E + 01	1.05E + 01	1.18E + 01
	DMSO	3.69E + 01	1.17E + 01	1.19E + 01	3.71E + 01	1.06E + 01	1.20E + 01
400	Gas phase	1.69E + 01	5.95E + 00	6.42E + 00	1.69E + 01	5.98E + 00	6.36E + 00
	water	2.21E + 01	7.73E + 00	8.23E + 00	2.22E + 01	7.76E + 00	8.26E + 00
	ethanol	2.11E + 01	7.43E + 00	7.92E + 00	2.12E + 01	7.46E + 00	7.93E + 00
	acetone	1.90E + 01	7.06E + 00	7.73E + 00	1.91E + 01	7.09E + 00	7.74E + 00
	DMSO	1.85E + 01	7.60E + 00	7.81E + 00	1.85E + 01	7.14E + 00	7.82E + 00

From Table 8, at *T = 250 K* and *P  = 1 atm*, the total unimolecular rate constants are 6.30 × 10^3^, 2.15 × 10^4^, 1.66 × 10^4^, 1.43 × 10^4^, and 1.50 × 10^4^s^− 1^ in gas phase, water, ethanol, acetone, and DMSO, respectively. In *T = 400 K*, the obtained results increase gradually to become 6.42, 8.23, 7.92, 7.73, and 7.81 s^− 1^, respectively.

All elementary forward and reverse reactions show a positive temperature dependence, with total rate constants increasing progressively as the temperature rises. The computed rate constants further reveal that the reverse reactions proceed significantly faster than their corresponding forward counterparts. Among the various decomposition pathways, the transformation of **CCPC** into structure C is identified as the most kinetically favorable route, likely attributed to its relatively low activation energy barrier. In addition, the rates of **CCPC** conversions are partially higher in water and acetone mediums relative to other mediums. In general, the role of tunneling becomes significant at low temperatures. The contributions of Eckert correction are high for the forward and reverse reactions of the *(R→****A****)* reaction compared to *(R→****B****)* and *(R→****C****)* reactions.

#### UV–Vis spectral analysis

**Fig. 7:**
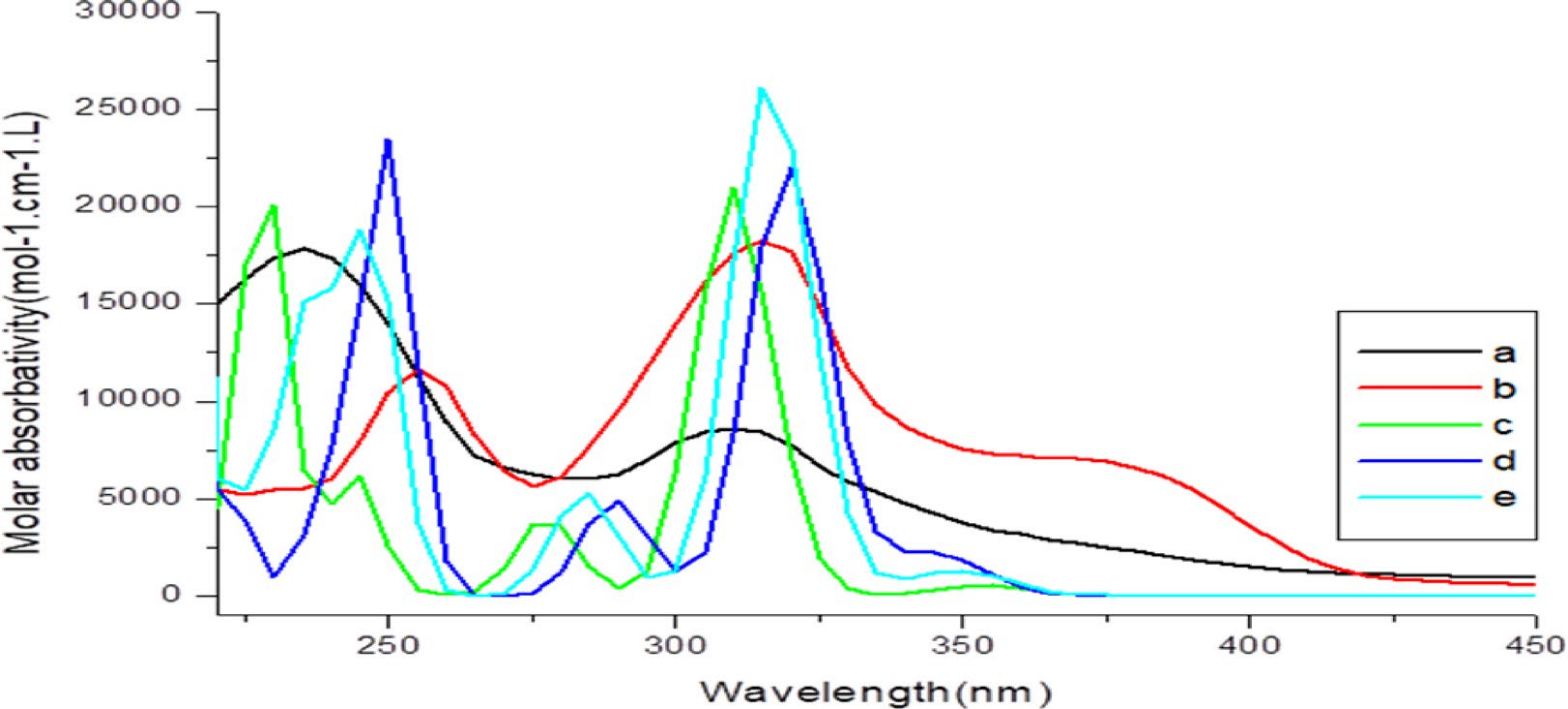
Electronic absorption spectra of **CCPC (a)** experimental in ethanol, **(b)** experimental in dioxane **(c)** theoretical in gas phase **(d)** theoretical in ethanol, **(e)** theoretical in dioxane. The concentration of the solute in (all UV) is 2.036 × 10^− 4^ mol/L in polar solvent and 1.131 × 10^− 4^ mol/L in non-polar solvent.

The principal electronic transitions contributing to the UV–visible absorption spectrum of **CCPC** were identified through theoretical calculations and are summarized in Table 9. Figure 7 compares the experimental and simulated UV–visible spectra. As shown in Table 9, the computed absorption wavelengths in the gas phase were observed at 300, 230, 210, 200, and 190 nm, corresponding to energy gaps of 4.35, 5.65, 5.90, 6.09, and 6.40 eV, respectively. The associated oscillator strengths for these transitions were 0.2273, 0.1352, 0.4707, 0.1418, and 0.2892, indicating the intensity and probability of each electronic excitation.

Based on the oscillator strength and absorption coefficient values, only the first transition exhibits significant absorption intensity, indicating that the band at 300 nm possesses the highest intensity, primarily attributed to a HOMO→LUMO transition with a 93% contribution. In ethanol, the calculated absorption wavelengths were 290, 260, 220, 210, and 195 nm, corresponding to energy gaps of 4.33, 4.85, 5.80, 5.85, and 6.30 eV, respectively. The corresponding oscillator strengths were 0.2416, 0.1769, 0.8833, 0.1536, and 0.2894. Notably, the first transition in ethanol also exhibited the most substantial HOMO→LUMO contribution at 96%, indicating it is the most dominant electronic excitation under these conditions.

**Table 9:** Experimental and Computed excitation energies (in eV), electronic transition configurations, and oscillator strengths ^a^ (ƒ) for the optical transitions of the absorption bands in the UV-vis. regions (involving HOMOs) of the compound **CCPC** at the CAM-B3LYP/6-311 + + G (d, p).

Medium	Transition	Excitation energies	Type of transition	$\:{\boldsymbol{\lambda\:}}_{\boldsymbol{m}\boldsymbol{a}\boldsymbol{x}/\mathbf{n}\mathbf{m}}$ $\:\boldsymbol{T}\boldsymbol{h}\:\boldsymbol{a}\boldsymbol{n}\boldsymbol{d}\:$ Ex.	Oscillator strengths (ƒ)	Configuration composition corresponding transition orbital
**Gas phase**	S0—> S1	4.35	n-π*	300	0.2273	-0.21 (49 ->51); -0.16 (49 ->53); 0.64 (50 ->51)
	S0—> S2	5.65	π-π*	230	0.1352	-0.26 (49 ->51); 0.40 (50 ->52); 0.43 (50 ->53); 0.12(50 ->55); -0.12(50 ->56)
	S0—> S3	5.90	π-π*	210	0.4707	-0.20 (47 ->51); 0.21 (49 ->52); 0.54 (49 ->53); 0.17 (50 ->51); 0.16 (50 ->54); 0.11 (50 ->56)
	S0—> S4	6.09	π-π*	200	0.1418	0.19 (47 ->51); 0.16 (47 ->53); 0.13 (49 ->53); 0.20 (50 ->53); 0.58 (50->56)
	S0—> S5	6.40	π-π*	190	0.2892	0.61 (47 ->51); -0.23 (49 ->53); -0.17 (50 ->56)
**Ethanol**	S0—> S1	4.33	n-π*	290 360	0.2416	-0.16 (49 ->51); 0.11 (49 ->52); -0.13 (49 ->53); 0.65 (50 ->51)
	S0—> S2	4.85	n-π*	260 310	0.1769	0.63 (49 ->51); 0.15 (50 ->51); -0.16 (50 ->52); 0.19 (50 ->53)
	S0—> S3	5.80	π-π*	220 235	0.8833	0.16 (49 ->51); -0.25 (49 ->52); 0.31 (49 ->53); 0.14 (50 ->51); 0.36 (50 ->52); -0.13 (50 ->53)
	S0—> S4	5.85	π-π*	210	0.1536	0.10 (45 ->51); 0.17 (49 ->52); 0.31(49 ->53); -0.23 (50 ->52); 0.38 (50 ->55); -0.13 (50 ->56)
	S0—> S5	6.30	π-π*	195	0.2894	0.46 (47 ->51); -0.22 (48 ->51); 0.15 (49 ->52) -0.22 (49 ->53); -0.36 (50 ->56)
**Dioxane**	S0—> S1	4.38	n-π*	295 380	0.2779	-0.18(49 ->51); -0.14(49 ->53); 0.65 (50 ->51)
	S0—> S2	4.91	n-π*	265 315	0.1882	0.61(49 ->51); 0.18(50 ->51); 0.26(50 ->53)
	S0—> S3	5.78	π-π*	225 255	0.5596	-0.27(49 ->51); -0.12(49 ->53); 0.20(50 ->52); 0.58(50 ->53)
	S0—> S4	5.90	π-π*	215	0.1979	0.38(49 ->53); -0.11(49 ->54); -0.35(50 ->52); 0.18(50 ->53); -0.19(50 ->55); 0.21(50 ->56)
	S0—> S5	6.36	π-π*	200	0.2654	0.59(47 ->51); -0.20(49 ->53); 0.11(49 ->56); 0.26(50 ->56)

The absorption wavelength of the first singlet excited state (S₀→S₁) in the gas phase is calculated to be 300 nm, with oscillator strength of 0.2273. Comparable absorption bands are observed at 290 nm and 295 nm in ethanol and dioxane, respectively, with corresponding oscillator strengths of 0.2416 and 0.2779. The increase in solvent polarity leads to a red shift in the absorption wavelength, indicating stabilization of the excited state in more polar environments. Experimentally, the UV-Vis absorption bands for the S₀→S₁ transition in ethanol and dioxane appear at 360 nm and 380 nm, respectively, demonstrating good agreement between theoretical predictions and experimental observations.

Additionally, other singlet excited states were examined following the same approach. It was found that increasing solvent polarity generally induced a blue shift in the absorption wavelengths. The observed transitions, primarily of the n→π* type, involve HOMO→LUMO excitation in both the gas phase and solvents. In the gas phase, the second singlet excited state (S₀→S₂) appeared at 230 nm, while in ethanol and dioxane; it was observed at 260 and 265 nm, respectively. These calculated values align reasonably well with the experimental absorption bands located at 310 and 315 nm in ethanol and dioxane, respectively. As solvent polarity increases from dioxane to ethanol, the spectral bands corresponding to both ground and excited states converge. Moreover, the intensity of these bands enhances in more polar solvents, indicating a shift in the nature of the transitions from n→π* to π→π*.

The UV/Vis absorption spectra of **CCPC** were theoretically computed for all singlet excited states (S₀ → S₅) in both the gas phase and two solvent environments, as summarized in Table 9. In general, the calculated data show strong correlation with the experimental UV-Visible spectra. Detailed analysis of the electronic transitions was carried out to assess the distribution of electron density across the contributing molecular orbitals. The identified absorption bands exhibit characteristics of localized excitations, delocalized π→π* transitions, and notable charge-transfer (CT) behavior.

## Conclusions

A new heterocyclic compound, 5-cyanomethylchromeno[4,3-b]pyridine (**CCPC**, **3**), was synthesized via a domino reaction, and a plausible reaction mechanism was proposed. Density functional theory (DFT/B3LYP/6-311 + + G(d, p)) calculations were employed to obtain optimized geometries, frontier molecular orbitals, and molecular electrostatic potential (MEP) maps, which helped rationalize the predicted reactive sites and provided theoretical support for the proposed pathway. The experimental IR and NMR spectra were found to be consistent with the calculated data, supporting the assigned structure. In silico ADME predictions suggest that **CCPC** possesses physicochemical properties compatible with drug-likeness criteria; however, these predictions require further experimental validation. Natural bond orbital (NBO) analysis indicated intramolecular charge delocalization contributing to electronic stabilization. Thermo-kinetic investigations revealed solvent-dependent variations in reaction rates, and TDDFT calculations suggested solvent-influenced electronic transitions in the simulated UV–Vis spectra. While the combined experimental and computational results provide supportive evidence for the structural characterization and electronic properties of **CCPC**, the study is limited to theoretical modeling and in silico pharmacokinetic assessment. Further experimental investigations, including extended mechanistic studies and biological evaluation, are necessary to validate the predicted reactivity patterns and assess potential practical applications.

## Supplementary Information

Below is the link to the electronic supplementary material.

Supplementary Material 1

## Data Availability

Yes, availability of Data and Materials. The datasets used and/or analyzed during the current study available from the corresponding author on reasonable request.

## References

[1] Kumar, S. et al. Advances in chromone-based copper(II) Schiff base complexes: synthesis, characterization, and versatile applications in pharmacology and biomimetic catalysis. *RSC Adv.* **14**, 17102. 10.1039/d4ra00590b (2024). 38808245 10.1039/d4ra00590b PMC11130647

[2] Abdelshafi, N. S. et al. Multifunctional Novel Lanthanide Complexes Based on Chromone Moiety for Corrosion Inhibition, Molecular Docking, and Anticancer and Antimicrobial Applications. *Appl. Organomet Chem*. **39**, e70009. 10.1002/aoc.70009 (2025).

[3] Guarneros-Cruz, K. A., Cruz-Gregorio, S., Romero-Ibañez, J., Meza-León, R. L. & Sartillo-Pisc, F. Synthetic Approach to Chromone and Flavonoid Piperidine Alkaloids. *J. Org. Chem.* **89**, 15808–15821. 10.1021/acs.joc.4c01926 (2024). 39437173 10.1021/acs.joc.4c01926 PMC11536364

[4] Ying, Z. et al. Two new chromones from Portulaca oleracea L. and their bioactivities. *Nat. Prod. Res.* **in press**10.1080/14786419.2024.2447053 (2025). 10.1080/14786419.2024.2447053 39749406

[5] Liu, X. et al. Anti-inflammatory 5,6,7,8-tetrahydro-2-(2-phenylethyl) chromone derivatives from the stems of Aquilaria sinensis. *Fitoterapia* **180**, 106312. 10.1016/j.fitote.2024.106312 (2025). 39603468 10.1016/j.fitote.2024.106312

[6] Qin, M. et al. Synthesis and anti-inflammatory activity of chromone-sulfonamide derivatives as COXs/iNOS dual-target inhibitors. *Med. Chem. Res.* **34**, 638–647. 10.1007/s00044-024-03368-z (2025).

[7] Husain, A., Anupama, B., Shaik, A. & Begum, A. Affinities and Antimicrobial Activities of Pd(II) Complexes of Chromone Schiff Bases. *App Organomet. Chem.* **39**, e70095. 10.1002/aoc.70095 (2025).

[8] Krishna, M. S. A. et al. Dual-acting β-Aminothiochromones: Design, synthesis, and evaluation as antimicrobial and anti-angiogenic agents. *Bioorg. Med. Chem. Lett.* **120**, 130140. 10.1016/j.bmcl.2025.130140 (2025). 39971201 10.1016/j.bmcl.2025.130140

[9] Kanzouai, Y. et al. Al Houari, Design, synthesis and characterization of new amide-linked Chromone-Isoxazole Hybrids: In Vitro anti-bacterial and antioxidant evaluation, DFT calculations, ADMET profiling, docking and molecular dynamics simulation. *J. Mol. Struct.* **1325**, 140972. 10.1016/j.molstruc.2024.140972 (2025).

[10] Serry, A. M. et al. In vitro and in vivo antidiabetic evaluation of new Coumarin and Chromone derivatives: Design, synthesis and molecular modeling. *Bioorg. Chem.* **159**, 108338. 10.1016/j.bioorg.2025.108338 (2025). 40101577 10.1016/j.bioorg.2025.108338

[11] Gu, Y., Qin, W., Xu, H. & Liu, Y. G. A simple chromone-derived fluorescent Turn–on probe for accurate detection of Al^3+^ Ions: Applications in food Analysis, test strips and bioimaging. *Spectrochim Acta A*. **329**, 125583. 10.1016/j.saa.2024.125583 (2025). 10.1016/j.saa.2024.125583 39689549

[12] Chumak, A. Y. et al. Thiazolic analogs of 3-OH-chromone: Synthesis, molecular structure, fluorescence spectra and photophysics. *J. Mol. Struct.* **1328**, 141357. 10.1016/j.molstruc.2025.141357 (2025).

[13] Al-Thani, A. F. A. A., Ba-Busail, F. E. A., Fadaly, M. K. S., Shraim, A. M. & Salih, K. S. M. Spectral, TD-DFT, and metal sensing investigations of four chromone-based compounds. *J. Mol. Struct.* **1325**, 141029. 10.1016/j.molstruc.2024.141029 (2025).

[14] Abdel-Megid, M., Badran, A. & Ibrahim, M. A. Synthetic routes for novel annulated chromeno[3,2:5,6]pyrido[2,3-*d*]imidazo[1,2-*a*]pyrimidines: Design, characterization, antimicrobial efficiency and theoretical studies. *J. Mol. Struct.* **1339**, 142347. 10.1016/j.molstruc.2025.142347 (2025).

[15] Mostafa, M. A., Ibrahim, M. A. & Badran, A. Spectroscopic elucidation, quantum chemical computations (FMO, HOMO–LUMO, MEP, NLO), and biological activity on some novel heterocyclic compounds using 3-substituted-6,8-dimethylchromones. *Synth. Commun.* **54** (18), 1523–1550. 10.1080/00397911.2024.2394833 (2024).

[16] Ibrahim, M. A. et al. Nucleophilic Reactions with 3-formylchromones: A decade update. *Synth. Commun.* **54** (18), 1495–1522. 10.1080/00397911.2024.2387134 (2024).

[17] Badran, A. et al. Ring opening and recyclization reactions with 3-functionalized chromones: Recent synthetic approaches for five, six and seven membered heterocycles. *Synth. Commun.* **55** (10), 693–716. 10.1080/00397911.2025.2463603 (2025).

[18] Elangovan, N. et al. Synthesis, structural analysis, and antimicrobial properties of (E)-2-((4-fluorobenzylidene) amino) phenol: A combined experimental and computational study. *J. Indian Chem. Soc.* **102** (5), 101659. 10.1016/j.jics.2025.101659 (2025).

[19] Elangovan, N. et al. Synthesis, spectroscopy, solvation effect, topology and molecular docking studies on 2,2′-((1,2 phenylenebis (azaneylylidene)) bis (methaneylylidene)) bis(4-bromophenol). *J. Mol. Struct.* **1322**, 140468. 10.1016/j.molstruc.2024.140468 (2025).

[20] Necmi, D. et al. Quantum computational, spectroscopic investigations on N-(2-((2-chloro-4,5-dicyanophenyl) amino) ethyl)- 4-methylbenzenesulfonamide by DFT/TD-DFT with different solvents, molecular docking and drug-likeness research. *Colloids Surf. A*. **638**, 128311. 10.1016/j.colsurfa.2022.128311 (2022).

[21] Abdulridha, A. A. et al. Corrosion inhibition of carbon steel in 1 M H_2_SO_4_ using new Azo Schiff compound: Electrochemical, gravimetric, adsorption, surface and DFT studies. *J. Mol. Liq*. **315**, 113690. 10.1016/j.molliq.2020.113690 (2020).

[22] Balakit, A. A. et al. Synthesis, spectrophotometric and DFT studies of new Triazole Schiff bases as selective naked-eye sensors for acetate anion. *Supramol Chem.* **32**, 519–526. 10.1080/10610278.2020.1808217 (2020).

[23] Soliman, H. N. & Yahia, I. S. Synthesis and technical analysis of 6-butyl-3-[(4-chlorophenyl)diazenyl]-4-hydroxy-2H-pyrano[3,2-*c*]quinoline-2,5(6*H*)-dione as a new organic semiconductor: Structural, optical, and electronic properties. *Dyes Pigm.* **176**, 108199. 10.1016/j.dyepig.2020.108199 (2020).

[24] Farag, A. A. M., Ibrahim, M. A., El-Gohary, N. M. & Roushdy, N. Synthesis, and photoelectrical characterizations of ECPPQT for optoelectronic application. *Arab. J. Chem.* **12**, 3723–3731. 10.1016/j.arabjc.2016.01.002 (2019).

[25] Abdel Halim, S. & Ibrahim Synthesis, M. A. DFT calculations, electronic structure, electronic absorption spectra, natural bond orbital (NBO) and nonlinear optical (NLO) analysis of the novel 5-methyl-8H-benzo[h]chromeno[2,3-b][1,6] naphthyridine-6(5H),8-dione (MBCND), J. Mol. Struct. 1130 543–558. (2017). 10.1016/j.molstruc.2016.10.058

[26] Ibrahim, M. A., Badran, A., El-Gohary, N. M. & Hashiem, S. H. Studies on the Chemical Reactions of Some 3-Substituted-6,8-dimethylchromones with Nucleophilic Reagents. *J. Heterocycl. Chem.* **55**, 2315–2324. 10.1002/jhet.3291 (2018).

[27] El Bakri, Y. et al. A highly substituted isoquinolinethione: Synthesis, crystal structure, DFT analysis and molecular docking studies against a series of the SARS-CoV-2 proteins. *J. Mol. Struct.* **1331**, 141527. 10.1016/j.molstruc.2025.141527 (2025).

[28] Celik, S. DFT investigations and molecular docking as potent inhibitors of SARS- CoV-2 main protease of 4-phenylpyrimidine. *J. Mol. Struct.* **1277**, 134895. 10.1016/j.molstruc.2022.134895 (2023). 36619799 10.1016/j.molstruc.2022.134895 PMC9803264

[29] Shalini, V. et al. Unveiling the structural and theoretical properties of 6-(2-fluoro-3-methylpyridin-4-yl)-2-(4-methoxyphenyl)-N-phenylquinoline-4-carboxamide compound as Sonic Hedgehog protein inhibitor: Synthesis, SCXRD, HSA, DFT, Docking and ADMET studies. *J. Mol. Struct.* **1330**, 141495. 10.1016/j.molstruc.2025.141495 (2025).

[30] Frisch, M. J. et al. (2009). J. Cioslowski, D.J. Fox, D. 0109, Revision D. 01, Gaussian, Inc., Wallingford, CT.

[31] Dennington, R., Keith, T. & Millam, J. GaussView, 5.0. 8; Gaussian Inc., (2008).

[32] Wolinski, K., Hinton, J. F. & Pulay, P. Efficient implementation of the gauge-independent atomic orbital method for NMR chemical shift calculations. *J. Am. Chem. Soc.* **112**, 8251–8260. 10.1021/ja00179a005 (1990).

[33] Das, S., Shedge, S. V. & Pal, S. Critical study of the charge transfer parameter for the calculation of interaction energy using the local hard-soft acid-base principle. *J. Phys. Chem. A*. **117**, 10933–10943. 10.1021/jp407070h (2013). 24066610 10.1021/jp407070h

[34] Avci, D. Second and third-order nonlinear optical properties and molecular parameters 8 of azo chromophores: semiempirical analysis. *Spectrochim Acta A*. **82**, 37–43. 10.1016/j.saa.2011.06.037 (2011). 10.1016/j.saa.2011.06.037 21835686

[35] Günay, N., Pir, H., Avcı, D. & Atalay, Y. NLO and NBO analysis of sarcosine maleic acid by using HF and B3LYP calculations. *J. Chem.* **712130**, 1–16. 10.1155/2013/712130 (2013).

[36] Tomasz, S. & Katarzyna, S. Benoît Ab initio Hartree-Fock calculations on linear and second-order nonlinear optical properties of ionic organic crystals. *Chem. Phys.* **141**, 104109. 10.1063/1.4894483 (2014). 10.1063/1.4894483 25217906

[37] Canneaux, S., Bohr, F. & Henon, E. KiSThelP: a program to predict thermodynamic properties and rate constants from quantum chemistry results. *J. Comput. Chem.* **35**, 82. 10.1002/jcc.23470 (2014). 24190715 10.1002/jcc.23470

[38] Abdel-Rahman, M. A., Shibl, M. F. & Mahmoud, M. A. M. Pyrolytic elimination of ethylene from ethoxyquinolines and ethoxyisoquinolines: A computational study. *Sci. Rep.* **13**, 6248. 10.1038/s41598-023-33272-2 (2023). 37069216 10.1038/s41598-023-33272-2 PMC10110564

[39] Eckart, C. Te penetration of a potential barrier by electrons. *Phys. Rev.* **35**, 1303–1309. 10.1103/PhysRev.35.1303 (1930).

[40] Reed, A. E., Weinstock, R. B. & Weinhold, F. Natural population analysis. *J. Chem. Phys.* **831** (10), 1736–154104. 10.1063/1.449486 (1985).

[41] Lee, C. T., Yang, W. T. & Parr, R. G. B. Development of the Colle-Salvetti correlation-energy formula into a functional of the electron density. *Phys. Rev.* **37**, 785–790. 10.1103/PhysRevB.37.785 (1988). 10.1103/physrevb.37.785 9944570

[42] Petersson, D. A. & Allaham, M. A. A complete basis set model chemistry. II. Open-shell systems and the total energies of the first‐row atoms. *J. Chem. Phys.* **94**, 6081–6090. 10.1063/1.460447 (1991).

[43] Khalaf, M. M. et al. Synthesis, characterization, DFT, biological activity evaluation, and molecular docking analysis of new 8-[(2-hydroxynaphthalen-1-yl) diazenyl] naphthalene-1, 3-disulfonic acid based complexes. *J. Mol. Struct.* **1300**, 137175. 10.1016/j.molstruc.2023.137175 (2024).

[44] Ramasamy, S. S. et al. In-Silico exploration: Unraveling the anti-cancer potential of 8-Nitroquinoline hydrazides. *J. Mol. Struct.* **1321**, 140218. 10.1016/j.molstruc.2024.140218 (2025).

[45] Kucuk, C., Celik, S., Yurdakul, S. & Coteli, E. A new Ag(I)-complex of 5-chloroquinolin-8-ol ligand: Synthesis, spectroscopic characterization, and DFT investigations, in vitro antioxidant (DPPH and ABTS), α-glucosidase, α-amylase inhibitory activities with protein-binding analysis. *J. Mol. Struct.* **1325**, 141285. 10.1016/j.molstruc.2024.141285 (2025).

[46] Alshaye, N. A., Ibrahim, M. A. & Badran, A. Nucleophilic transformation of 3-substituted-6,8-dimethylchromones with phenylhydrazine under various reaction conditions: Theoretical, Spectroscopic characterization and in silico ADME studies. *J. Mol. Struct.* **1297**, 37006. 10.1016/j.molstruc.2023.137006 (2025).

[47] Abd El-Lateef, H. M., Ali, A. M., Khalaf, M. M. & Abdou, A. New iron (III), cobalt (II), nickel (II), copper (II), zinc (II) mixed-ligand complexes: Synthesis, structural, DFT, molecular docking and antimicrobial analysis. *Bull. Chem. Soc. Ethiop.* **38**, 147–166. 10.4314/bcse.v38i1.12 (2024).

[48] Abd El-Lateef, H. M., Khalaf, M. M., Heakal, F. E. T. & Abdou, A. Fe (III), Ni (II), and Cu (II)-moxifloxacin-tri-substituted imidazole mixed ligand complexes: Synthesis, structural, DFT, biological, and protein-binding analysis. *Inorg. Chem. Commun.* **158**, 111486. 10.1016/j.inoche.2023.111486 (2023).

[49] Parr, R. G. & Pearson, R. G. Absolute hardness: companion parameter to absolute electronegativity. *J. Am. Chem. Soc.* **105**, 7512–7516. 10.1021/ja00364a005 (1983).

[50] Abd El-Lateef, H. M., Khalaf, M. M., Amer, A. A., Abdelhamid, A. A. & Abdou, A. Antibacterial, antifungal, anti‐inflammatory evaluation, molecular docking, and density functional theory exploration of 2‐(1*H*‐benzimidazol‐2‐yl) guanidine mixed‐ligand complexes: Synthesis and characterization. *Appl. Organomet. Chem.* **38**, e7299. 10.1002/aoc.7299 (2024).

[51] Gul, S. et al. Exploring the synthesis, molecular structure and biological activities of novel Bis-Schiff base derivatives: A combined theoretical and experimental approach. *J. Mol. Struct.* **1306**, 137828. 10.1016/j.molstruc.2024.137828 (2024).

[52] Sert, Y., Albayati, M. R., Şen, F. & Dege, N. The DFT and in-silico analysis of 2, 2′-((1e, 1′ e)-((3, 3′-dimethyl-[1, 1′-biphenyl] – 4, 4′ diyl) bis (azanylylidene)) bis (methanylylidene)) diphenol molecule. *Colloids Surf. A*. **687**, 133444. 10.1016/j.colsurfa.2024.133444 (2024).

[53] Elkotamy, M. S. et al. Novel imidazo[2,1-*b*]thiazoles and imidazo[1,2-*a*] pyridines tethered with indolinone motif as VEGFR-2 inhibitors and apoptotic inducers: Design, synthesis and biological evaluations. *Bioorg. Chem.* **151**, 107644. 10.1016/j.bioorg.2024.107644 (2024). 39079394 10.1016/j.bioorg.2024.107644

[54] Matta, C. F. Modeling biophysical and biological properties from the characteristics of the molecular electron density, electron localization and delocalization matrices, and the electrostatic potential. *J. Comput. Chem.* **35**, 1165–1198. 10.1002/jcc.23608 (2014). 24777743 10.1002/jcc.23608 PMC4368384

[55] Omer, R. A., Ahmed, K. M., Omar, K. A., Hamad, W. M. & Mamad, D. M. N, N-Bis (2, 4-dihydroxy benzaldehyde) benzidine: Synthesis, characterization, DFT, and theoretical corrosion study. *J. Mol. Struct.* **1300**, 137279. 10.1016/j.molstruc.2023.37279 (2024).

[56] Suja, R. et al. Synthesis, spectroscopic analysis (FT-IR, NMR), non-covalent interactions (RDG, IGM) and dynamic simulation on Bis (8-hydroxyquinoline)salicylate salicylic acid, *J. Mol. Struct.* **1310,** 138231. (2024). 10.1016/j.molstruc.2024.138231

[57] Bazrafshan, M. et al. Synthesis, molecular structure, conformational, and intramolecular hydrogen bond strength of ethyl 3-amino-2-butenoate and its N-Me, N-Ph, and N-Bn analogs; An experimental and theoretical study. *J. Mol. Struct.* **1274**, 134479. 10.1016/j.molstruc.2022.134479 (2023).

[58] Daina, A., Michielin, O. & Zoete, V. SwissADME: a free web tool to evaluate pharmacokinetics, drug-likeness and medicinal chemistry friendliness of small molecules. *Sci. Rep.* **7**, 42717. 10.1038/srep42717 (2017). 28256516 10.1038/srep42717 PMC5335600

[59] Pogaku, V., Gangarapu, K., Basavoju, S., Tatapudi, K. K. & Katragadda, S. B. Design, synthesis, molecular modelling, ADME prediction and anti-hyperglycemic evaluation of new pyrazole-triazolopyrimidine hybrids as potent α-glucosidase inhibitors. *Bioorg. Chem.* **93**, 103307. 10.1016/j.bioorg.2019.103307 (2019). 31585262 10.1016/j.bioorg.2019.103307

[60] Sures, S. The Growth and the Optical, Mechanical, Dielectric and Photoconductivity Properties of a New Nonlinear Optical Crystal—L-Phenylalanine-4-nitrophenol NLO Single Crystal. *J. Crystallization Process. Technol.* **3**, 87–91. 10.4236/jcpt.2013.33014 (2013).

[61] Cheng, L. T. et al. Electric field induced second harmonic generation with and without fringes. *J. Phys. Chem.* **95**, 10631 10.11648/j.ijctc.20190701.19/JPCHAX

[62] Kaatz, P., Donley, E. A. & DP Shelton Analysis of nonlinear optical properties in donor–acceptor materials. *J. Chem. Phys.* **108**, 849. 10.1063/1.475448 (1998). Citation, CAS. 10.1063/1.4874267 24832271

[63] Gnanasambandan, T., Gunasekaran, S. & Seshadri, S. Experimental and theoretical study of *p*-nitroacetanilide. *Spectrochim Acta A*. **117**, 557–567. 10.1016/j.saa.2013.08.061 (2014). 10.1016/j.saa.2013.08.061 24036264

[64] J. Chocholousova, V. Spirko and P. Hobza, first local minimum of the formic acid dimer exhibits simultaneously red-shifted O–H… O and improper, blue-shifted C–H….O hydrogen bonds, *Phys. Chem*. **6**, 37–41. (2004) 10.1039/B314148A

[65] Szafran, M., Komasa, A. & Bartoszak-Adamska, E. Crystal and molecular structure of 4-carboxypiperidinium chloride (4-piperidinecarboxylic acid hydrochloride). *J. Mol. Struct.* **827**, 101–107. 10.1016/j.molstruc.2006.05.012 (2007).

