# Supplementary material for: Experimental, spectroscopic, thermodynamic, and DFT study of a novel cyanomethylchrome nopyridinecarbonitrile (CCPC)
Source: Sci Rep. 2026 Mar 27;16:10899. doi: 10.1038/s41598-026-41126-w (PMC13039783; doi:10.1038/s41598-026-41126-w)
Supplement: Supplementary file 1 — Supplementary Material 1 [file 41598_2026_41126_MOESM1_ESM.docx]

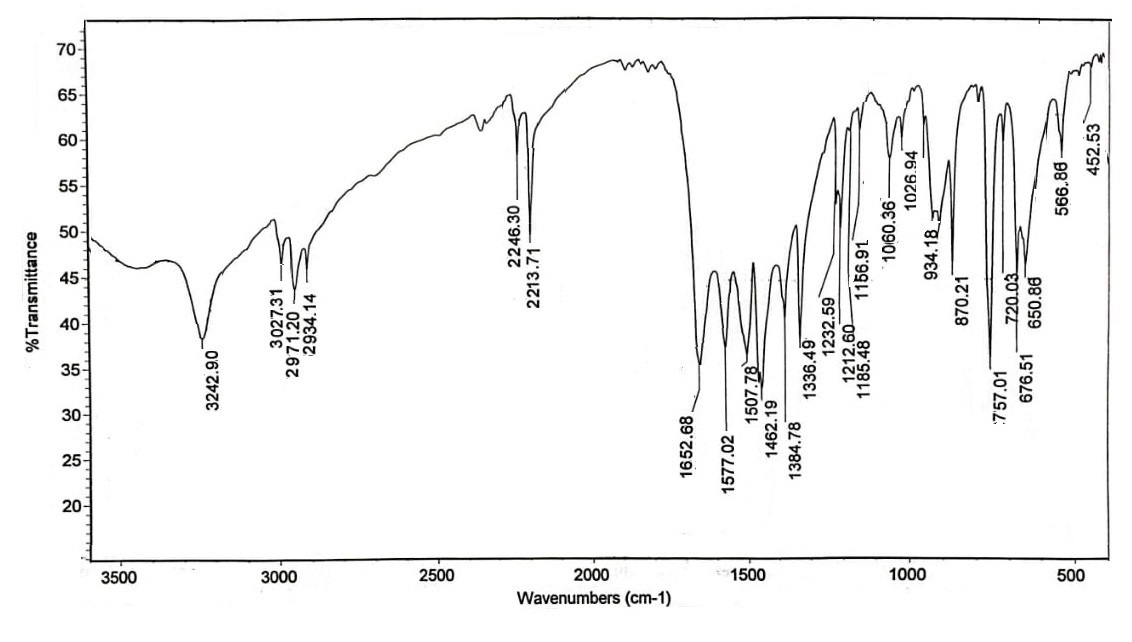

**Figure S1:** (a) Experimental and (b) Calculated IR spectra of compound **3** (**CCPC**) at B3LYP/6-311++G(d,p).


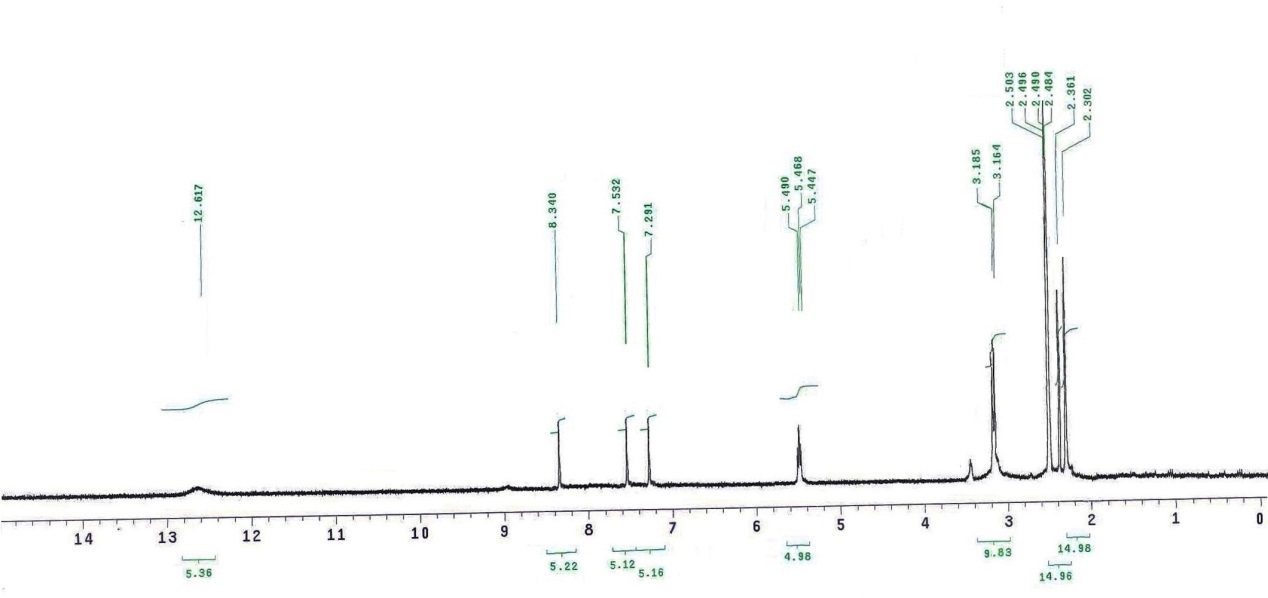

**Figure S2**: (a) Experimental and (b) Calculated ^1^H NMR spectra of compound **3** (**CCPC**) at B3LYP/6-311++G(d,p).


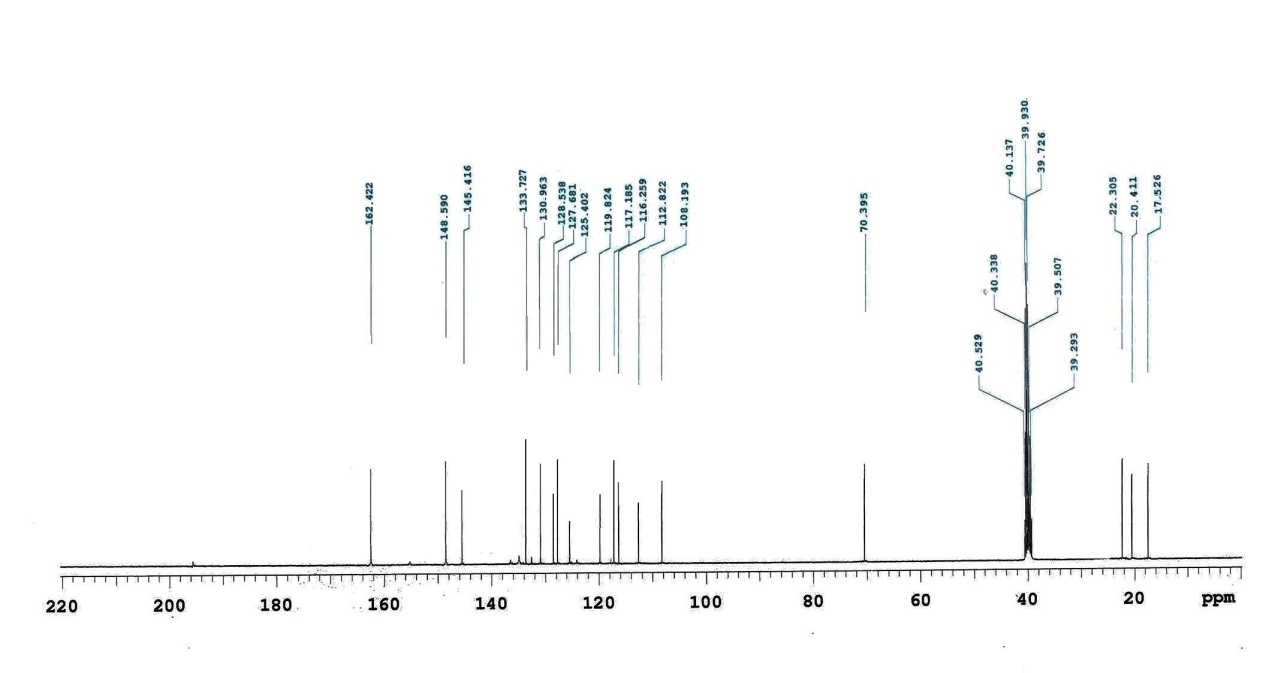

**Figure S3:** (a) Experimental and (b) Calculated ^13^C NMR spectra of compound **3** (**CCPC**) at B3LYP/6-311++G(d,p).


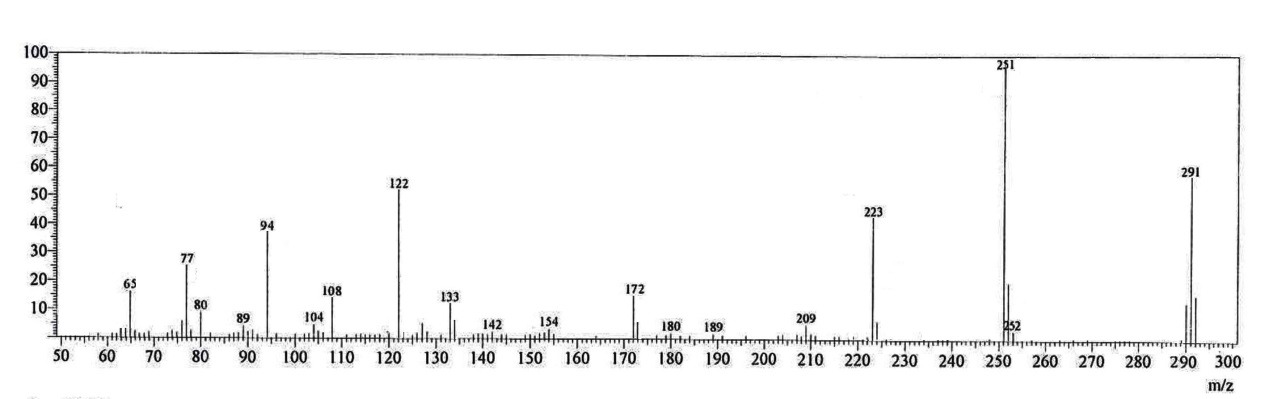


**Figure S4:** Mass spectrum of compound **3** (**CCPC**)
